# Are different populations fairly represented in single-cell omic atlases?

**DOI:** 10.1016/j.xgen.2026.101300

**Published:** 2026-07-20

**Authors:** Catrina Yang, Kavitharini Saravanan, Aryan Saharan, Kuan-lin Huang

**Affiliations:** 1 University of Oxford, Green Templeton College, Medical Sciences Division, Oxford OX2 6HG, UK; 2 University of North Carolina at Charlotte, Charlotte, NC, USA; 3 Saint Louis University, St. Louis, MO, USA; 4 Department of Genetics and Genomic Sciences, Department of Artificial Intelligence and Human Health, Center for Transformative Disease Modeling, Tisch Cancer Institute, Icahn Genomics Institute, Icahn School of Medicine at Mount Sinai, New York, NY 10029, USA

## Abstract

Single-cell omic atlases are transforming biology and medicine, yet their demographic representativeness has not been systematically evaluated. We analyzed >13,500 samples from the Human Cell Atlas (HCA), Human Tumor Atlas Network (HTAN), and PsychAD Consortium. Benchmarking against global and US general and disease-prevalence data, we found a striking, pervasive European overrepresentation and underrepresentation of Asian and Latino individuals. Nearly 70% of HCA samples lacked ancestry annotation, and among annotated samples, Europeans were overrepresented 6-fold. PsychAD was nearly two-thirds European. HTAN tumors were 69% European, with several cancer types showing sex skews beyond expected incidence. These disparities highlight that current single-cell resources risk embedding inequities into AI foundational models, biomarker discovery, and therapeutic development. We contextualize these findings within the structural, economic, and regulatory spaces; survey the growing ecosystem of diversity-focused initiatives; and provide an actionable, field-specific checklist to help research teams design single-cell studies whose benefits extend equitably across populations.

## Graphical abstract

**Figure:**
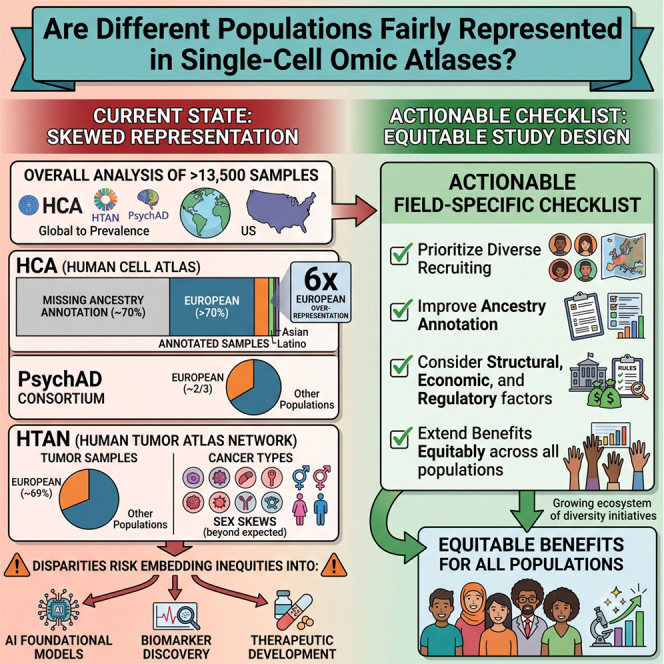

## Teaser

Single-cell atlases serve as a foundational resource for biomedical discovery and AI foundation models. But who is represented in those atlases? Yang et al. audit three widely used atlases—the Human Cell Atlas, the HTAN, and PsychAD—and find that European donors are overrepresented relative to individuals of other ancestries, and in several cases, ancestry/sex metadata remain unannotated. A 16-item checklist is provided to design equitable single-cell studies and ensure equitable benefits from the resulting scientific advances.

## Introduction

Single-cell omics technologies have revolutionized our understanding of cellular heterogeneity, enabling the discovery of rare cell types, transitional states, and microenvironmental dynamics that bulk approaches obscure. Large-scale initiatives such as the Human Cell Atlas (HCA),^1^^,^^2^^,^^3^ the Human Tumor Atlas Network (HTAN),^4^^,^^5^ and the PsychAD Consortium^4^^,^^6^ have generated expansive datasets spanning healthy tissues, tumor microenvironments, and neuropsychiatric or neurodegenerative conditions. These resources are invaluable to biomedical discovery, but systematic evaluation of their demographic composition has been limited, raising concerns about their representativeness and generalizability.

The challenge of representation has long plagued human genomics. Genome-wide association studies (GWASs) have been overwhelmingly conducted in individuals of European ancestry, leading to biased polygenic risk scores (PRSs) that transfer poorly to other populations.^7^^,^^8^ PRSs derived from European cohorts often lose their predictive accuracy when applied to non-European groups.^9^^,^^10^ Beyond causing technical issues for research, the lack of diversity in genomics risks reinforcing health disparities when biomedically actionable findings are disproportionately relevant to majority populations.^11^

Genetic ancestry shapes cell-type-specific gene expression and regulatory architecture. Single-cell studies of viral immune responses have identified hundreds of genes with ancestry-differential expression in specific immune cell subsets,^12^^,^^13^ and patient ancestry modulates innate immune activation at the level of individual cell states in SARS-CoV-2 responses.^14^ An atlas from one ancestral background therefore risks missing cell subtypes or wrongly assuming universal cell markers—paralleling how the original reference genome missed variants or genome segments now recovered by the Human Pangenome Project.^15^ While definitive population-specific cell types or cell states remain to be proven, many pathogenic variants are unique to specific populations,^16^^,^^17^ and ancestry-driven regulatory variation can shift cell states in disease-relevant cell populations that a homogeneous atlas would fail to catalog. Diverse single-cell maps are therefore a prerequisite for biological completeness—a *pancellular* reference that, like the pangenome, must represent humanity’s full diversity to be scientifically universal.^18^^,^^19^

**Figure 1:**
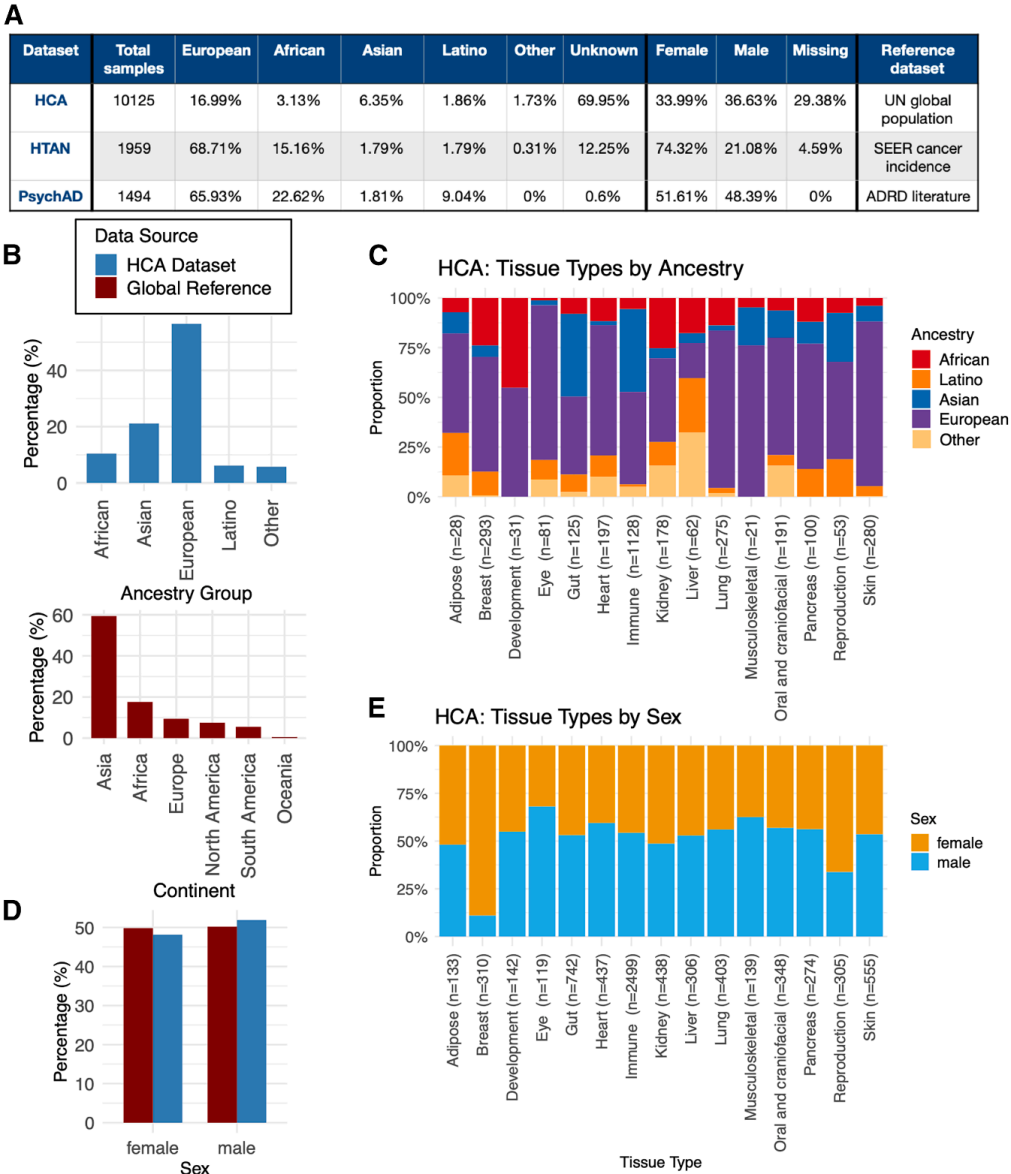
Overview of example atlases and Human Cell Atlas ancestry and sex composition across tissues (A) Overall ancestry and sex composition of individuals from the Human Cell Atlas (HCA), the Human Tumor Atlas Network (HTAN), and the PsychAD consortia evaluated in this study. (B) Comparison of ancestry proportions in the HCA (excluding samples with unknown ancestries, in red bars), with proportions of European, African, Asian, Latino, and other ancestry to the global reference data of populations across continents (in blue bars). (C) Stacked barplots of ancestry distributions across 15 HCA tissue categories (excluding samples with unknown ancestries), with proportions of European, African, Asian, Latino, other, and unknown ancestry shown for each tissue. (D) Comparison of sex proportions in the HCA (excluding samples with unknown sexes, in red bars) to global population reference distributions (in blue bars). (E) Stacked barplots of sex distributions across 15 HCA tissue categories (excluding samples with unknown ancestries), with proportions of female and male shown for each tissue.

Single-cell consortia, where significant resources are unified to survey cellular profiles in thousands of individuals, risk reproducing inequities in genomics. The outputs of these large-scale studies are often marketed as universal reference maps of human biology and used to train foundation models.^2^^,^^20^^,^^21^^,^^22^ However, recent studies have suggested that the profiled cellular omics signatures could indeed be ancestry and sex specific,^18^^,^^19^ calling for the need to establish single-cell datasets that fairly represent different populations. Here, we systematically evaluate ethnicity/race and sex representations of sampled individuals in three major single-cell consortium-generated atlases (Figure 1A), providing a diversity audit to inform future study designs.

## HCA vs. global population demographics

The HCA dataset comprised 10,125 samples across 15 tissue categories,^1^^,^^2^ the largest included immune (*n* = 3,704, 36.6%), gut (*n* = 883, 8.7%), skin (*n* = 906, 8.9%), lung (*n* = 743, 7.3%), and kidney (*n* = 601, 5.9%) (Figure S1; Table S1). Overall, 69.9% of samples lacked ancestry annotation after we thoroughly cataloged data from the HCA website or supplemental information reported by the linked publications (methods). Among the 3,043 samples with reported ancestry, Europeans comprised 56.5%, followed by Asians (21.1%), Africans (10.4%), Latinos (6.2%), and other (5.8%) (Figure 1B). Compared to global expectations (Europe: 9.4%, Asia: 59.4%, Africa: 17.6%, and Latino: 13.6%), Europeans were overrepresented 6-fold, while Asians, Africans, and Latinos were consistently underrepresented (0.36×, 0.59×, and 0.45×, respectively; *p* < 2e−16). We acknowledge that the use of the global reference is an oversimplification and provides only coarse categories that align across studies following the extensive curation of a wide range of ancestry metadata (methods).

Because conclusions drawn from complete-case analysis alone could be severely biased if missingness is non-random, we considered a simple thought experiment of how the observed European share would change at two extremes: if every unlabeled donor turned out to be European (pushing the share to its highest possible value, or ceiling) vs. if none of them were European (pushing it to its lowest possible value, or floor). The true share has to lie somewhere between these two extremes, regardless of why annotations are missing. This approach is known as partial-identification bounds, where the upper bound (ceiling) assumes that all missing donors belong to the group of interest and the lower bound (floor) assumes that none do (Table S2). While other ancestries’ underrepresentation becomes less stable under conservative missingness estimates, European overrepresentation is robust to all plausible missing-data mechanisms: even under the most conservative assumption (all 7,082 missing donors are non-European), the European lower bound (17.0%) still exceeds the global reference (9.4%).

Tissue-level analyses revealed pronounced but heterogeneous skews—all tests compared observed ancestry mix within tissue to the global reference mix (see methods) showed a false discovery rate (FDR) < 0.01, except for musculoskeletal, where 205/226 were of unknown ancestry (Figure 1C). Among the ancestry-labeled samples most biased for European samples, skin was 82.9% European, lung 79.3%, eye 77.8%, musculoskeletal 76.2%, and heart 65.5%. By contrast, immune and gut showed comparatively higher Asian representation (41.7% and 41.6%, respectively), with lower European fractions (immune: 46.5% and gut: 39.2%). African-ancestry individuals were better represented in development (45.2%), kidney (25.3%), and breast (23.9%) atlases, whereas Latino individuals showed higher representation in liver (27.4%), adipose (21.4%), and reproduction (18.9%) atlases (Figure 1C). These skews in ancestral proportions likely reflect study-purpose-driven sampling rather than intentional exclusion: many HCA datasets derive from tissue-banking programs with non-random recruitment (organ donors, surgical excess, and pediatric samples) whose demographics reflect the population served by contributing institutions. We note that high ancestry missingness in the HCA (69.9%) likely obscures the true ancestry compositions and should be addressed in future releases.

Sex ratios in the HCA were 34.0% female, 36.6% male, and 29.4% unreported (Figures S1 and 1D). Among samples with sex information, expected skews were observed in breast (89.0% female) and reproductive (66.2% female) tissues. Several tissues were modestly male enriched, including immune (54.3% male), eye (68.1% male), lung (56.1% male), and musculoskeletal. No significant sex biases were observed for adipose, development, gut, kidney, and liver (*p* > 0.17), suggesting more balanced sampling in these tissue atlases (Figure 1E). These sex patterns are sometimes directionally consistent with physiological expectations in sex-specific disease tissues but also suggest potential ascertainment or study-design bias elsewhere.

## The HTAN vs. the US cancer population incidence

The HTAN dataset comprised 1,959 tumor samples across 22 cancer types.^4^^,^^5^ Breast cancer was the largest contributor (*n* = 993, 50.7%), followed by lung (*n* = 245, 12.5%), colorectal/colon (*n* = 151, 7.7%), skin (*n* = 79, 4.0%), neuroblastoma (*n* = 74, 3.8%), pancreas (*n* = 47, 2.4%), ovary (*n* = 26, 1.3%), and several smaller categories (<20 cases each) (Figure S2; Table S3). Overall, 12.3% of samples lacked ancestry annotation. Among the 1,719 samples with reported ancestry, Europeans comprised 78.3%, Africans 17.3%, Asians 2.0%, Latinos 2.0%, and other 0.3%.

**Figure 2:**
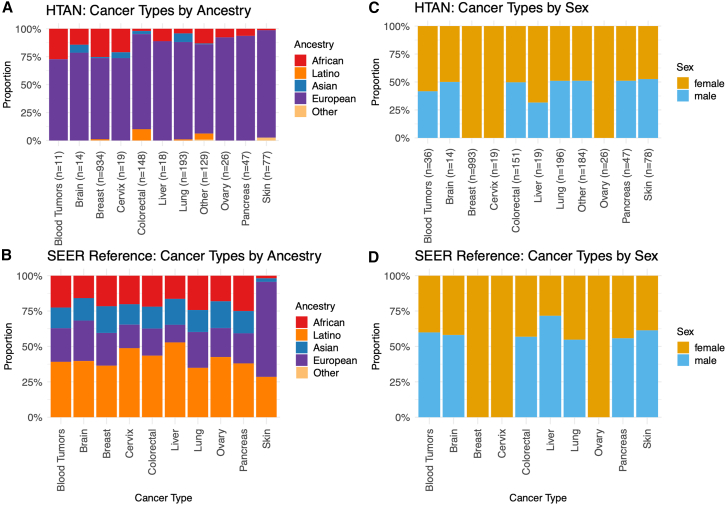
Human Tumor Atlas Network ancestry and sex composition across cancer types (A and B) Stacked barplots comparing ancestry distributions across 11 major cancer categories in (A) the Human Tumor Atlas Network (HTAN; excluding samples with unknown ancestry) vs. (B) the reference SEER cancer incidence data of the respective cancer type, as shown in proportions of European, African, Asian, Latino, other, and unknown ancestry. (C and D) Stacked barplots comparing sex proportions in (C) the HTAN (excluding samples with unknown sex) with (D) the reference SEER cancer incidence data of the respective cancer type.

Cancer-specific ancestry distributions revealed additional skew in several cancer types relative to their expected US incidences (Figures 2A and 2B). In samples with annotated ancestry data, all cancer tissue sites showed over 72% European ancestry. For example, skin cancer showed 96.1% European ancestry, pancreas 93.6%, ovary 92.3%, liver 88.9%, and lung 87.1%. Substantial African representation was found in blood tumors (27.3%), breast (25.3%), cervix (21.1%), and neuroblastoma (17.8%). Asian and Latino participants were severely underrepresented in all cancer tissue sites; the highest count of Asian participants was in lung (*n* = 15/245), which comprised 7.8% of the cohort, whereas the highest count of Latino participants was found in colorectal cancer (*n* = 15/151) (Figures 2A and 2B).

The overall sex distribution was heavily female (74.3% female, 21.1% male, and 4.6% unreported) (Figure S2), driven by the predominance of breast cancer that comprised over half the cohort and minorly by the other tissue sites, i.e., ovary and cervix, that were female specific. Considering only cases with reported sex, multiple cancer types showed a female skew in the HTAN, such as liver (68.4% female), lung (49%), blood (58.3%), and skin (47.4%) tumors, compared with the Surveillance, Epidemiology, and End Results (SEER) female/male incidence of those respective cancers (Figures 2C and 2D). These sex patterns are sometimes directionally consistent with physiological expectations in sex-specific disease tissues but also suggest potential ascertainment or study-design bias elsewhere.

## The PsychAD Consortium vs. US demographics in neurological diseases

The PsychAD dataset comprised 1,494 samples drawn from three contributing cohorts: Harvard Brain Tissue Resource Center (HBCC; *n* = 300), Mount Sinai NIH Brain Bank (MSBB; *n* = 1,042), and Rush Alzheimer’s Disease Center (RADC; *n* = 152).^4^^,^^6^ Across the cohort, diagnostic categories included Alzheimer’s disease (AD_combined; *n* = 373, 25.0%), schizophrenia (SCZ; *n* = 120, 8.0%), dementia with Lewy bodies (DLBD; *n* = 112, 7.5%), vascular dementia (*n* = 85, 5.7%), bipolar disorder (BD; *n* = 55, 3.7%), tauopathies (*n* = 47, 3.1%), Parkinson’s disease (PD; *n* = 40, 2.7%), frontotemporal dementia (FTD; *n* = 15, 1.0%), and dementia not otherwise specified (*n* = 439, 29.4%); in PsychAD, some individuals may have multiple diagnoses, and 597 individuals do not have any of these diagnoses (Figure S3; Table S4).

**Figure 3:**
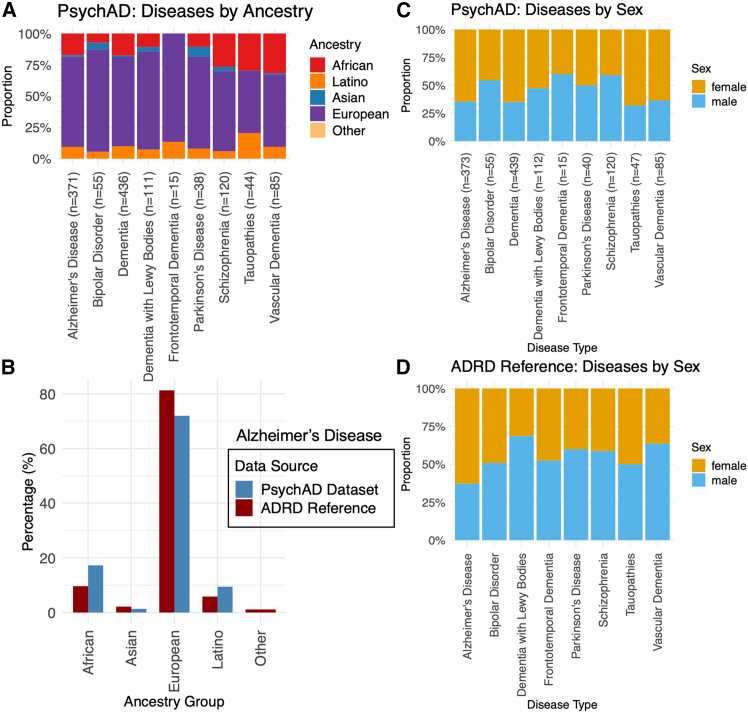
PsychAD ancestry and sex composition across neuropsychiatric and neurodegenerative diagnoses (A) Stacked barplots of ancestry distributions across 9 diagnostic categories, including Alzheimer’s disease, schizophrenia, dementia with Lewy bodies, vascular dementia, bipolar disorder, tauopathies, Parkinson’s disease, frontotemporal dementia, and dementia not otherwise specified. (B) Comparison of ancestry proportions in PsychAD Alzheimer’s disease cases (excluding samples with unknown ancestries, in red bars), with proportions of European, African, Asian, Latino, and other ancestry to the reference data of US Alzheimer’s disease and related dementia cases (in blue bars). (C and D) Stacked barplots comparing sex proportions in (C) PsychAD (excluding samples with unknown ancestries) with (D) the reference data from literature for each dementia type.

Among all 1,494 Psych samples, Europeans accounted for 65.9%, Africans 22.6%, Latinos 9.0%, Asians 1.8%, and unknown 0.6%. Compared to AD and related dementia (ADRD) reference demographics in the US (Europeans: 59.1%, Africans: 12.2%, Latinos: 9.2%, and Asians: 6.6%) (methods) (Figures 3A and 3B), AD cases in PsychAD were characterized by overrepresentation of Europeans (71.6%), slightly higher representation of Africans (17.2%), and near-parity Latinos (9.4%) but marked underrepresentation of Asians (1.4%). For other diseases, Europeans are heavily represented in FTD (86.7%), BD (81.8%), and DLBD (77.7%). African individuals comprised a considerable fraction of vascular dementia (31.8%) and SCZ (26.7%) cases. The highest percentages of Latino individuals were found within tau (19.2%) and FTD (13.3%) cases. The highest fractions of Asian individuals were found in PD and BD, at merely 7.5% and 5.5%, respectively (Figures 3A and 3B).

Overall sex balance was near parity: 51.6% female and 48.4% male (Figure S3). Disease-level analyses revealed stronger deviations from equal representation, and disease-specific sex ratios were compared with their respective reference data (methods; Figures 3C and 3D). AD and dementia not otherwise specified were 64.6%/64.9% female, respectively, consistent with known female predominance. Vascular dementia also showed female enrichment in PsychAD (63.5% female). On the other hand, male skews were seen in SCZ (59.2% male, aligned with literature’s 58.7%), BD (54.6%), FTD (60%), and control (57.8%). While PD had 20 males and 20 females in PsychAD, the known disease incidence is about 60% male (Figures 3C and 3D).

### Significant ancestry and sex biases exist in surveyed single-cell atlases

Our systematic assessment across the HCA,^1^^,^^2^ the HTAN,^4^^,^^5^ and PsychAD^4^^,^^6^ shows that demographic bias is pervasive in single-cell atlases. The HCA had nearly 70% of ancestry annotations missing, and among labeled donors, European individuals dominated all tissue types. The HTAN revealed even starker distortions: Europeans comprised >78% of annotated samples and were overrepresented in every cancer type compared with SEER US cancer incidence, while Asians and Latinos were severely underrepresented in all cancer types. PsychAD was nearly two-thirds Europeans, with African ancestry better represented at 22.6% (higher than expected based on ADRD prevalence), but Asians were again underrepresented (1.8%). Sex balance was dataset dependent: near parity in PsychAD overall, male skewing in multiple HCA immune tissues, and female skewing in the HTAN across several non-sex-specific cancers.

These findings extend the well-documented inequities of genomics.^7^^,^^9^^,^^11^ Just as GWASs and PRSs suffer from limited transferability across ancestries,^8^^,^^10^ single-cell resources biased toward European donors risk producing cell and disease maps that fail to generalize globally. This is not a trivial concern: genetic background influences gene expression and regulatory variation,^23^ sex differences shape immune responses and disease trajectories,^24^ and ancestry-associated biology can alter therapeutic options and responses; e.g., in genetics, *CYP2D6* allele frequencies governing metabolism of ∼25% of clinical drugs differ markedly across populations.^25^^,^^26^ While single-cell signatures have yet to be widely used for clinical stratification, overrepresentation of Europeans in single-cell genomics precludes the discovery of biology that exists across the wide spectrum of human diversity and risks building single-cell models that obscure the cellular mechanisms most relevant to underrepresented groups.

This study has several limitations. First, ancestry was analyzed as reported metadata rather than genetically inferred, which may introduce misclassification. Classifying ancestry groups into broad categories such as European or Asian is challenging due to the complex and personal nature of ethnic identity. These broad categories collapse substantial within-group heterogeneity and conflate self-identified race/ethnicity with genetic ancestry. Where metadata permitted, we sought finer granularity (see methods: curation of demographic data in single-cell consortium studies), but most datasets did not support it. Some groups, such as Ashkenazi Jews, classified as European, have distinct cultural and genetic backgrounds that may not fit neatly into this category. Similarly, Hispanic/Latino encompasses individuals with mixed heritage. Second, the high degree of missing data limits resolution, and small sample sizes for certain groups reduced statistical power for some disease-specific comparisons. Third, our analyses of ancestral/sex proportions do not resolve whether the imbalances result from study-purpose-driven sampling or intentional/unintentional exclusion. Fourth, the use of reference populations herein is limited by their surveyed populations and time points; for example, the global demographics of continental population shares used for the HCA will not match genetic ancestry estimates. Finally, our analyses focused on 3 publicly available single-cell consortium datasets, which may not reflect the demographic composition of all other single-cell omic resources. Non-consortium datasets collectively have exceeded these consortia in sample size and may differ in composition but often offer even less standardized metadata for analyses. Conclusions herein are based on data from these specific consortia, not the field as a whole. Despite these caveats, the consistency of European overrepresentation and Asian/Latino underrepresentation across these datasets underscores the robustness of our conclusions.

### How can we fix this disparity?

The implications of disparity in single-cell omics are both scientific and societal. Scientifically, biased datasets constrain the interpretability of findings, hinder reproducibility, and limit the scope of discovery. Missing metadata compounds these problems, as incomplete annotation disproportionately affects careful study designs that account for diversity and limits the interpretability of cross-population analyses. Societally, they risk exacerbating health disparities by embedding inequities into the reference frameworks that guide biomarker discovery, drug development, and precision medicine. This can lead to challenges when a significant portion of future advances may be driven by AI foundation models built on these skewed datasets. For example, single-cell AI foundation models that are already widely used, such as scGPT,^20^ Geneformer,^27^ and scFoundation,^28^ maximized the size of their training datasets by combining existing public datasets. Consequently, most foundation models include the HCA as one of the major sources and leverage HTAN data when they separately trained a cancer-focused model without, to our knowledge, supplementation of data from underrepresented populations,^20^^,^^21^^,^^22^ thus possibly inheriting the bias from these datasets without most users’ awareness. A controlled benchmark of AI model performance stratified by donor ancestry remains needed. Moreover, most AI tools are developed at Western institutions with GPU infrastructure; researchers in low- and middle-income countries may lack resources to run these models, creating a secondary inequity whereby analytical tools—not just reference data—reflect a narrow perspective. Providing technology access programs in both single-cell and AI technologies is needed to help address such disparity.

The demographic skews emerge from intersecting structural forces. Most atlas projects draw samples from readily available sources at major institutions, often defaulting to local demographics unless diversity is considered during study design. Funding for large-scale projects has historically come from wealthier countries without explicit diversity requirements. Large research institutions that conduct such studies may also concentrate on certain geographical regions where recruited samples do not reflect the diversity of national or global populations. Single-cell technology remains expensive, limiting participation from low-resource settings. National data governance policies create additional constraints: many countries restrict sharing individual-level genomic data outside their borders. Finally, achieving equity requires respectful community engagement—many populations have historical or cultural reasons for caution, and frameworks such as Indigenous data sovereignty principles^29^ appropriately constrain how data can be shared.

The research community is actively addressing these gaps. The HCA Equity Working Group and Diversity Task Force track and encourage donor diversity reporting.^30^ In 2021, Chan Zuckerberg Initiative (CZI) launched Ancestry Networks for the HCA funding single-cell data collection from underrepresented populations worldwide, supporting projects including African, Latin American, and Asian immune diversity atlases and ancestrally inclusive organ atlases.^31^ A recent framework from the HCA outlines governance, inclusive funding, and cross-regional partnership principles for equitable consortia.^1^ Technically, initiatives to estimate genetic ancestry directly from single-cell RNA sequencing (scRNA-seq) data via single-nucleotide-polymorphism-based approaches^32^ may provide ancestry estimates for nearly all atlas samples, superseding reliance on self-reported labels. Data portals, including CELLxGENE and the European Bioinformatics Institute (EBI) Single Cell Expression Atlas (SCEA), are incorporating structured ancestry fields in metadata schemas.

Moving forward, several structural reforms are still needed. First, intentional recruitment strategies should align donor sampling with disease incidence and population demographics,^33^ as proposed in recent roadmaps for equity in genomics.^9^ Second, metadata completeness and standardization should be mandated in single-cell repositories, with ancestry, sex, and tissue/disease-specific clinical variables treated as required fields. This can be achieved by harmonized toolsets and checklists such as those provided by the HCA Ethics Working Group.^34^ Indeed, datasets such as PsychAD have much more complete metadata, suggesting this could be achieved with careful study and workflow design. Third, equity metrics should be adopted as benchmarks for evaluating single-cell datasets, complementing measures of resolution and scale with measures of demographic diversity.

To translate this into actionable steps, we developed a field-specific checklist (Table 1) covering 16 specific questions and 5 domains across the full study life cycle: procurement and recruitment planning aligned to sampling frame; pre-analytical considerations including tissue dissociation and donor availability; equitable multiplexing and batch-assignment strategies to avoid confounding demographics with batch effects; minimum required demographic metadata fields and controlled vocabularies (aligned with CELLxGENE and EBI standards); standardized biobanking protocols; consent language and data-sovereignty considerations for international collaborations; and AI model-development reporting standards including training-data composition disclosure, stratified performance evaluation across ancestry and sex, and robustness testing. We include the AI/machine learning (ML) model-development items within this single-cell checklist because cellular reference atlases and the AI foundation models trained on them are now produced as a continuous pipeline: the same study-design choices that determine donor diversity at recruitment propagate directly into the training corpora of downstream cell-state classifiers and foundation models. Embedding these considerations in a single checklist clarifies accountability. Each domain pairs a critical question with concrete solutions—for example, stratified recruitment quotas, community clinic partnerships, and pre-specified minimum sample sizes per ancestry group. This approach aims to address bias proactively, at the point of study design, where correction is most effective.

**Table 1:** Practical checklist for equitable single-cell omic study design

No.	Key question for research teams	Possible solutions
**Recruitment and study design**		

1	does the planned cohort reflect population incidence/prevalence for the target tissue/disease?	• set explicit sampling quotas by ancestry • oversample underrepresented groups
2	is sex balance planned for non-sex-specific tissues/diseases?	• set sex-stratified quotas
3	have we defined minimum per-group sample sizes to enable stratified analyses by ancestry and/or sex?	• power analyses by subgroup; enforce thresholds • phase enrollment until quotas are met
4	are community sites serving underrepresented populations included as recruitment hubs?	• add community clinics and advisory boards • culturally tailored outreach and consent
5	are eligibility criteria free of inadvertent exclusions (language, insurance, visit schedule)?	• translated materials and flexible visit windows • reimbursements/transport, childcare support

**Pre-analytical and single-cell specific**		

6	do tissue procurement and dissociation protocols account for donor availability constraints across sites (e.g., organ-donor programs, surgical schedules)?	• pre-qualify multiple procurement pipelines per site • standardize ischemia time, dissociation enzyme/duration, and viability thresholds
7	are biobanking standard operating procedures (SOPs) harmonized across sites (time to freeze, buffers, RNase control)?	• unified SOPs with site audits and quality control dashboards • reject out-of-spec samples uniformly
8	do multiplexing and batch-assignment strategies avoid confounding ancestry/sex with technical batch?	• randomize or block donors across pools/batches by ancestry and sex • include cross-site reference samples in every run • use equitable barcoding allocation (balance hashtag/lipid oligos across groups)

**Metadata and monitoring**		

9	will ancestry, sex, age, and disease-specific clinical variables be mandatory fields using controlled vocabularies?	• require fields at upload using ontology standards (e.g., CELLxGENE schema, EBI SCEA metadata) • validate against HAncestro for ancestry
10	are missingness dashboards (ancestry × sex × site) reviewed regularly?	• publish dashboards; open action items to sites when gaps emerge
11	is there a plan for post hoc enrichment if monitoring shows imbalance?	• pre-specify trigger rules (e.g., <20% of target quota) • add contingency recruitment sites; targeted outreach
12	will raw + harmonized metadata be versioned and publicly released?	• robust versioning and changelog on every data release

**Community engagement and ethics**		

13	have we planned return of value to sustain participation from diverse communities?	• share lay summaries and participant newsletters • host community feedback sessions
14	does consent language address data sovereignty, international sharing, and re-use in AI model training?	• consult local governance frameworks (e.g., CARE Principles for Indigenous Data) • include explicit clauses on computational re-use and AI model training

**Downstream AI/ML model development**		

15	will training-data demographic composition be disclosed in model documentation?	• report ancestry/sex breakdown of all training and test splits • flag known gaps relative to target application population
16	will model performance be evaluated and balanced when stratified by ancestry and sex?	• report cell-type annotation accuracy, embedding quality, and cell marker predictions per demographic group • include robustness tests under simulated demographic shift

Single-cell omics risks repeating inequities in genomic studies—with consequences extending to the cellular reference maps that define human biology. Unless corrective actions are taken at the level of study design, consortium governance, funding policy, and technical infrastructure, the resulting benefits may become unevenly distributed. The HCA Equity Working Group, CZI Ancestry Networks, and regional initiatives represent real progress. Our analysis provides a quantitative baseline across three major consortia and provides a framework against which this progress can be measured. A *pancellular* reference representing humanity’s full ancestral, sex, and environmental diversity is the only atlas that will deliver on single-cell biology’s translational promise for all populations.

## Methods

### Study design and data sources

We conducted a systematic evaluation of sex- and ancestry-related representation across three large-scale single-cell resources: the HCA, the HTAN, and the PsychAD Consortium. These datasets were chosen because they represent flagship community efforts to generate, harmonize, and disseminate high-resolution cellular and disease-relevant data. For contextual benchmarking, we compared observed representation in each dataset against appropriate reference distributions: (1) global ancestry proportions from United Nations (UN) population statistics for the HCA, (2) SEER cancer incidence rates stratified by ancestry and sex for the HTAN, and (3) ADRD prevalence estimates for PsychAD. Each of these data sources is described in detail later in the text.

In brief, metadata files from the HCA, the HTAN, and PsychAD were harmonized into canonical categories. Ancestry was standardized into African, Asian, European, Latino, other, and unknown. For the HTAN, Hispanic/Latino identifiers were mapped to Latino regardless of race; for PsychAD, genetic ancestry labels (e.g., European [EUR], African [AFR], admixed American [AMR], East Asian [EAS], and South Asian [SAS]) were collapsed into the canonical categories. Biological sex was standardized to female, male, or missing. For disease and tissue annotations, HCA samples were grouped by tissue of origin (15 categories), HTAN samples by cancer type (22 categories), and PsychAD samples by diagnosis (9 categories).

### Statistical analyses

We computed descriptive statistics for ancestry and sex distributions overall and within tissues or disease categories. Representation was quantified both as raw counts and as proportions among non-missing annotations. Chi-squared tests compared observed vs. expected representation using population-level baselines (global ancestry, SEER, or ADRD). Multiple testing corrections were further conducted using the Benjamini-Hochberg procedure for FDR. To measure magnitude of imbalances, we also calculated representation ratios (observed ÷ expected), equity indices (symmetrized measures of under- vs. overrepresentation), and diversity metrics (Simpson’s and Shannon’s indices) in the code-generated logs.

For the HCA, analyses were stratified by tissue type; for the HTAN, by cancer type; and for PsychAD, by diagnosis. Within each stratum, we assessed ancestry composition, sex distribution, and statistical significance relative to reference populations. We also quantified missingness for ancestry and sex. All analyses were performed in R using packages including *dplyr*, *tidyr*, *ggplot2*, *car*, and *scipy*. Scripts were version controlled, and all transformations were logged for reproducibility.

### Global reference population

We used the share of the world population by major world region (continent). Specifically, we used the widely cited 2021 distributions: Asia, 59.4%; Africa, 17.6%; Europe, 9.4%; North America, 7.5%; South America, 5.5%; and Oceania, 0.6%. These values correspond to UN-based tallies compiled in the “World population by continent, 2021” table.^35^ Global sex composition was derived from the UN World Population Prospects (WPP) 2022 summary, which reports that the world had 50.3% males and 49.7% females in 2022.^36^

### US cancer reference population

We used SEER∗Explorer (National Cancer Institute) to obtain age-adjusted incidence rates (per 100,000) for 2018–2022 by cancer site, race/ethnicity, and sex. SEER∗Explorer methods specify that rates are age adjusted to the 2000 US standard population and are reported per 100,000 persons; incidence estimates incorporate SEER delay-adjustment methodology.^37^

For ancestry/ethnicity, we used the five mutually exclusive SEER race/ethnicity categories:

• Non-Hispanic (NH) White.
• NH Black.
• NH American Indian/Alaska Native (AI/AN).
• NH Asian/Pacific Islander (API).
• Hispanic (any race).

These are standard, mutually exclusive SEER groupings (Hispanic supersedes race). Sex was coded as male and female.

SEER provides rates rather than percentage shares. To construct reference distributions (for ancestry and for sex) within each cancer site, we did the following:

(1)We retrieved age-adjusted incidence rates for the target grouping (e.g., five race/ethnicity groups or the two sex groups).
(2)For each cancer site, we converted rates to shares by dividing each group’s rate by the sum of rates across groups for that site (i.e., compositional normalization so that shares sum to 1.0 within the site).

These normalized shares were used as the US cancer reference for ancestry/ethnicity and sex composition, respectively. Because rates are age adjusted, the share is a proxy for the composition of incident cases, not general population composition.

### ADRD reference population

#### US ancestry/ethnicity composition (ADRD)

For ADRD, we used the analysis by Matthews et al., which reported age-standardized prevalence estimates by race/ethnicity in adults aged ≥65 years.^38^ Specifically, they reported the 2014 number of Medicare fee-for-service (FFS) beneficiaries with ADRD in each of the NH White, Black, API (annotated as Asian for our analysis), Hispanic (annotated as Latino for our analysis), and AI/AN and other (grouped into others for our analysis). For the other diseases, we could not find consistent race-specific incidence data and thus were not modeled.

#### Sex composition

To standardize sex differences in incidence and prevalence across neuropsychiatric and neurodegenerative diseases in the US, we relied on the best available population-based studies and large systematic reviews, converting reported sex-specific rates or ratios into fractions that sum to one. For AD and ADRD, we adopted the Global Burden of Disease 2019 estimate showing that globally in 2019, there were substantially more women than men living with dementia, with a female-to-male ratio of 1.69 (95% UI: 1.64–1.73).^39^ For BD, US National Comorbidity Survey data indicate nearly equal prevalence—2.8% in women vs. 2.9% in men—resulting in fractions of 0.491 and 0.509, respectively.^40^ DLBD is markedly male predominant: in Olmsted County, Minnesota, incidence was 4.8 per 100,000 person years in men compared with 2.2 in women, yielding fractions of 0.314 and 0.686.^41^ FTD overall shows little sex bias, with pooled case series reporting near parity (373 males vs. 338 females), translating to fractions of 0.525 and 0.475.^42^ PD consistently demonstrates male predominance in North America, with men approximately 1.5 times more likely than women to develop the disease, corresponding to fractions of 0.600 and 0.400.^43^ SCZ also occurs more frequently in men, with meta-analyses estimating a male-to-female incidence rate ratio of ∼1.42:1, equivalent to 0.587 male and 0.413 female fractions.^44^ Tauopathies as an aggregate entity are epidemiologically heterogeneous: progressive supranuclear palsy often shows modest male predominance, while corticobasal degeneration is closer to balanced; given this inconsistency, we reported a neutral 0.5/0.5 split as an aggregate reference.^45^ Finally, vascular dementia appears more common in men in high-income cohorts, with estimates such as 5.6 vs. 3.2 cases per 1,000 person years, corresponding to 0.636 male and 0.364 female fractions.^46^

### Curation of demographic data in single-cell consortium studies

#### HCA

##### Data source

We used publicly available datasets from the HCA Bionetwork^1^^,^^2^^,^^3^ to assess ethnic disparities in single-cell genomics research. The analysis covered datasets from 14 HCA networks, including adipose, breast, development, eye, gut, heart, immune, kidney, liver, musculoskeletal, nervous system, oral and craniofacial, pancreas, reproduction, and skin. Two networks, genetic diversity and organoid, did not have available datasets and were excluded from this analysis.

##### Dataset selection and metadata extraction

For each network, we accessed all available datasets to review the associated metadata files, typically provided in Excel spreadsheet format. These metadata files were examined to identify clinical and demographic information about the donors, specifically focusing on age, gender, ethnicity, and disease status. If demographic metadata were present, we extracted and compiled the relevant information into a single comprehensive spreadsheet for each network.

In cases where ethnicity data were absent from the metadata, we manually reviewed the publications associated with the dataset. These publications were linked within each dataset’s page and often contained supplemental information. Ethnicity data were most frequently found in the materials and methods sections or the supplemental materials of these publications. We documented the presence or absence of metadata for each dataset and categorized the ethnicity reporting as follows.

(1)Metadata availability: we recorded whether the study included metadata with clinical and demographic data.
(2)Ethnicity reporting in metadata: if ethnicity was present in the metadata, we marked it as reported.
(3)Ethnicity reporting in publications: if no ethnicity data were found in the metadata, we documented whether they were available in the publication.

If ethnicity was provided in the metadata, the column asking about ethnicity data in publications was marked as “N/A.” In cases where the ethnicity data in the publication were incomplete or unclear (e.g., presented as proportions or graphs without clear detail), we flagged this limitation with an asterisk.

##### Standardization of ancestry and ethnicity categories

Given the variability in how ethnicity data were reported across projects (e.g., Asian, East Asian, or Korean), we standardized the ethnicity categories to allow for consistent comparisons across datasets. The following broad categories were used.

• European.
• African.
• East Asian.
• South Asian.
• Asian: for cases where further specification was not provided.
• American: including Hispanic/Latino and Native American individuals.
• Other: encompassing other ethnic groups not covered by the categories above and individuals of mixed ethnic backgrounds.
• Unknown.

All Asian categories were eventually merged in analyses due to constraints from some studies. All more granular data were also preserved in the data available for download in this manuscript.

Donors from other species (e.g., *Mus musculus*) and cell lines, including human induced pluripotent stem cells (iPSCs), were excluded from the dataset.

#### HTAN

We analyzed HTAN^4^^,^^5^ data by filtering for datasets with scRNA-seq data. For each HTAN dataset, we accessed the associated metadata files and reviewed sample-level attributes including primary tissue of origin, tumor type, anatomical location, and contributing research center. To ensure consistency across datasets, we created a standardized labeling system for tissue origin and tumor site, modeled after the category structure used on the HTAN homepage. Where metadata fields were ambiguous or inconsistent, we manually reconciled them using information from the associated publications and supplemental materials. Each sample was then re-annotated using this uniform classification framework, allowing for improved comparison across atlases. Given the limited number of samples in certain cancer types, all common hematologic malignancies were assigned to a single blood tumors category, and bone/bone marrow/joint sites were merged to a bones category. The curated metadata were compiled into a master file that enables streamlined analysis of tissue-specific patterns and site-based tumor characteristics.

To classify ancestry groups, scRNA-seq metadata from HTAN were extracted and analyzed for racial/ethnic differences. For the purpose of this study, individuals reported as Hispanic/Latino were considered as Latino; individuals reported White but non-Latino or missing ethnicity were considered as European; individuals reported as African or African American were considered as African; individuals reported to be East Asian, South Asian, or EAS_SAS were considered Asian; individuals reported to be other values were considered as other; and unknown or missing data were considered as unknown.

#### PsychAD Consortium

We downloaded the metadata from supplemental table 1 of PsychAD’s latest data release and landmark manuscript^6^ as of May 2025. In these metadata, ancestries of AFR are mapped to African, AMR to Latino, EUR to Europeans, and EAS/SAS/EAS_SAS to Asians. To identify each sample’s disease status, we simply used the coded variables where an individual could have more than one diagnosis, and all others without any diagnosis are considered controls.

## Data and code availability

The curated metadata datasets for this study, including the HCA, the HTAN, and PsychAD and their reference datasets, are deposited in https://zenodo.org/records/17161565.

The source code to reproduce all analyses can be found at https://github.com/Huang-lab/scOmics_equity under the open-source MIT license.

## Acknowledgments

The authors wish to acknowledge the Dataworks! Prize hosted by the Federation of American Societies for Experimental Biology (FASEB) and the National Institutes of Health (NIH) for inspiring them to conduct this study. The study was based on volunteer efforts; the publication is funded by 10.13039/100000002NIH 10.13039/100000049NIA/10.13039/100000051NHGRI UG3AG105083 and 10.13039/100000057NIGMS R35GM138113 and 2R35GM138113 to K.-l.H.

## Author contributions

K.-l.H. conceived the research and designed the analyses. C.Y. curated the HCA data, A.S. curated the HTAN data, and K.S. conducted the exploratory analyses. K.-l.H. curated the PsychAD data and reference data. K.-l.H. conducted all final analyses and supervised the study. K.-l.H. wrote the manuscript, and all authors read, edited, and approved the manuscript.

## Declaration of interests

K.-l.H. is a co-founder and board member of 501(c)(3) not-for-profit Open Box Science and Kaimen, Inc., both of which were unrelated to this study.

## Supplemental information

Data S1. Tables S1–S4

Document S2. Transparent peer review records for Yang et al.

Document S3. Article plus supplemental information
