# Supplementary material for: Are different populations fairly represented in single-cell omic atlases?
Source: Cell Genom. 2026 Jul 20;6(8):101300. doi: 10.1016/j.xgen.2026.101300 (PMC13477034; doi:10.1016/j.xgen.2026.101300)
Supplement: Supplementary file 1 [file mmc1.pdf]

Cell Genomics, Volume *6*

Supplemental information

Are different populations fairly represented

in single-cell omic atlases?

Catrina Yang, Kavitharini Saravanan, Aryan Saharan, and Kuan-lin Huang

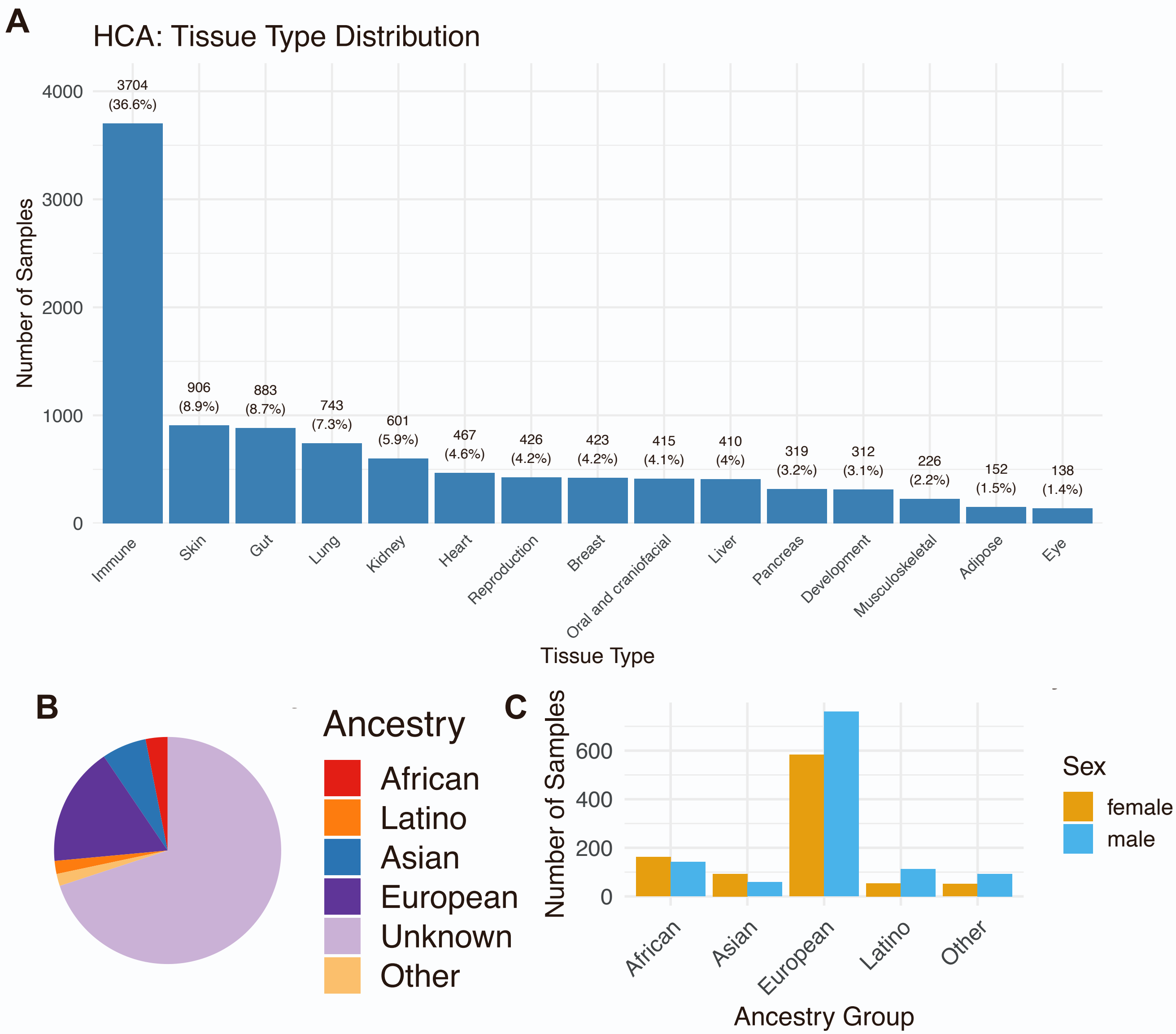
A
HCA: Tissue Type Distribution
Number of Samples
4000
3000
2000
1000
0
3704 (36.6%)
906 (8.9%)
883 (8.7%)
743 (7.3%)
601 (5.9%)
467 (4.6%)
426 (4.2%)
423 (4.2%)
415 (4.1%)
410 (4%)
319 (3.2%)
312 (3.1%)
226 (2.2%)
152 (1.5%)
138 (1.4%)
Immune
Skin
Gut
Lung
Kidney
Heart
Reproduction
Breast
Oral and craniofacial
Liver
Pancreas
Development
Musculoskeletal
Adipose
Eye
Tissue Type
B
Ancestry
African
Latino
Asian
European
Unknown
Other
C
Number of Samples
600
400
200
0
African
Asian
European
Latino
Other
Ancestry Group
Sex
female
male

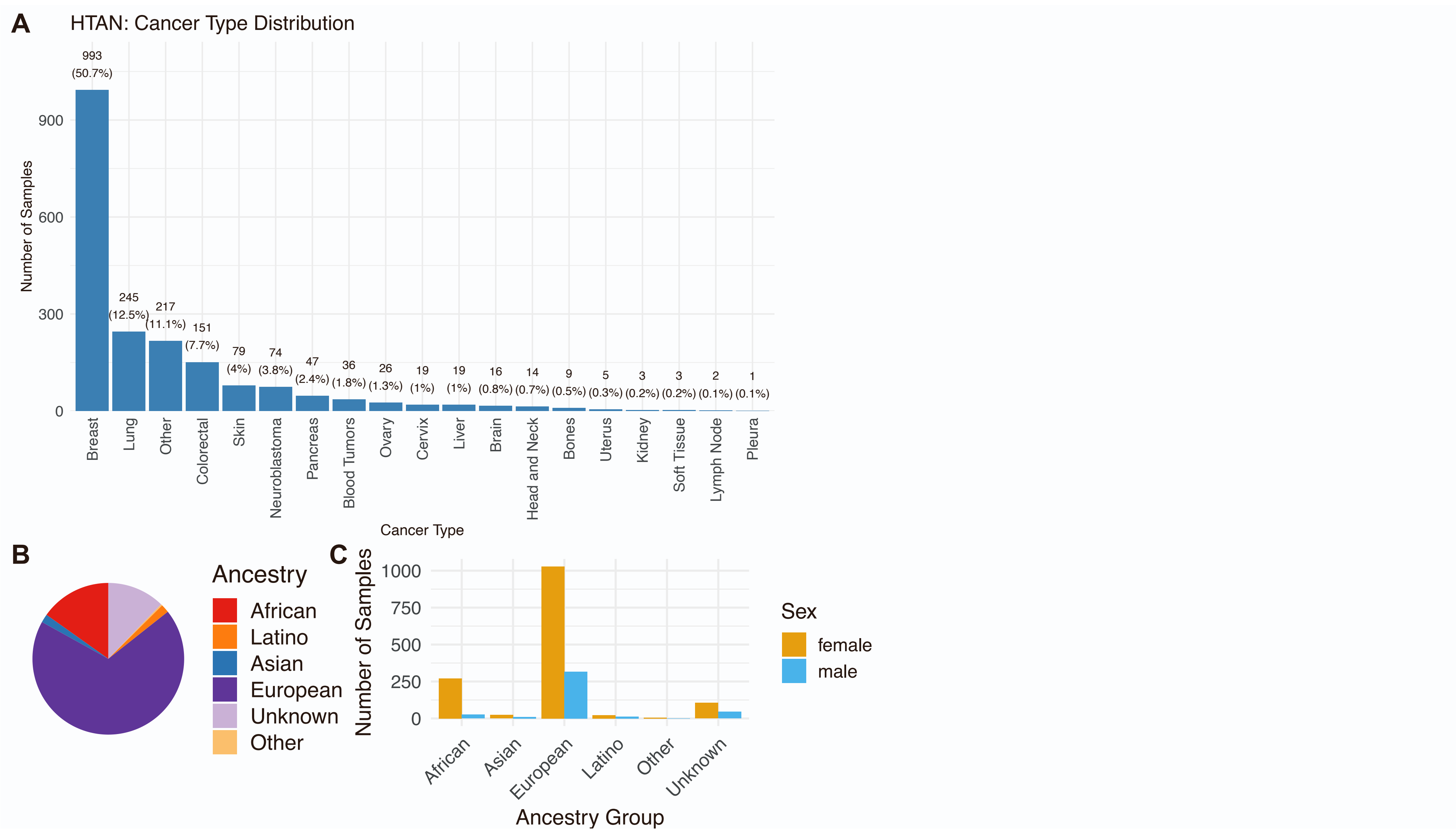
A
HTAN: Cancer Type Distribution
Number of Samples
Cancer Type
993 (50.7%)
245 (12.5%)
217 (11.1%)
151 (7.7%)
79 (4%)
74 (3.8%)
47 (2.4%)
36 (1.8%)
26 (1.3%)
19 (1%)
19 (1%)
16 (0.8%)
14 (0.7%)
9 (0.5%)
5 (0.3%)
3 (0.2%)
3 (0.2%)
2 (0.1%)
1 (0.1%)
Breast
Lung
Other
Colorectal
Skin
Neuroblastoma
Pancreas
Blood Tumors
Ovary
Cervix
Liver
Brain
Head and Neck
Bones
Uterus
Kidney
Soft Tissue
Lymph Node
Pleura
B
Ancestry
African
Latino
Asian
European
Unknown
Other
C
Number of Samples
Ancestry Group
African
Asian
European
Latino
Other
Unknown
Sex
female
male

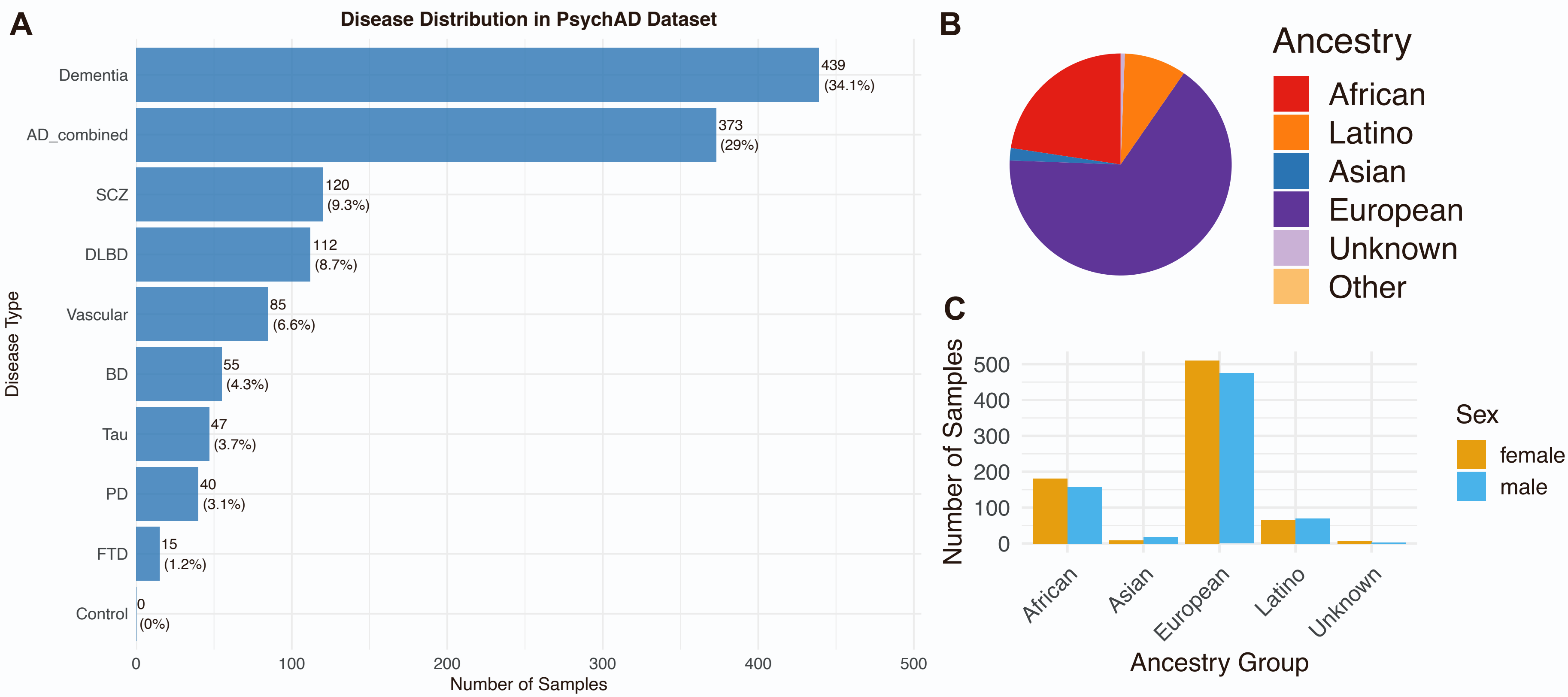
A
Disease Distribution in PsychAD Dataset
Dementia
AD_combined
SCZ
DLBD
Vascular
BD
Tau
PD
FTD
Control
439 (34.1%)
373 (29%)
120 (9.3%)
112 (8.7%)
85 (6.6%)
55 (4.3%)
47 (3.7%)
40 (3.1%)
15 (1.2%)
0 (0%)
Disease Type
0 100 200 300 400 500
Number of Samples
B
Ancestry
African
Latino
Asian
European
Unknown
Other
C
Number of Samples
0 100 200 300 400 500
African Asian European Latino Unknown
Ancestry Group
Sex
female
male
