# Supplementary material for: Are different populations fairly represented in single-cell omic atlases?
Source: Cell Genom. 2026 Jul 20;6(8):101300. doi: 10.1016/j.xgen.2026.101300 (PMC13477034; doi:10.1016/j.xgen.2026.101300)
Supplement: Supplementary file 2 [file mmc3.pdf]

Cell Genomics
Transparent Peer Review Record

Are Different Populations Fairly Represented in Single-Cell Omic Atlases?

Author list: Catrina Yang, Kavitharini Saravanan, Aryan Saharan, Kuan-lin Huang

Summary

Initial submission:	Received : November 5th 2025
	Scientific editor: Judith Nicholson
First round of review:	Number of reviewers: 3<br>Revision invited : February 23rd 2026<br>Revision received : March 18th 2026
Second round of review:	Number of reviewers: 2<br>Accepted : June 16th 2026
Data freely available:	Yes
Code freely available:	N/A

This transparent peer review record is not systematically proofread, type-set, or edited. Special characters, formatting, and equations may fail to render properly. Standard procedural text within the editor's letters has been deleted for the sake of brevity, but all official correspondence specific to the manuscript has been preserved.

Referees' reports, first round of review

Reviewer 1:

In this Perspective paper, the authors conduct a secondary analysis of publicly available metadata of large single-cell consortia, including HCA, HTAN, and PsychAD and argue that existing single-cell resources exhibit substantial ancestry and sex biases. The topic may be of broad interest, but in its current form, the manuscript suffers from conceptual oversimplification, methodological opacity, and overstatement of conclusions relative to the evidence provided. Substantial revision and strengthening are required before this work could be suitable for publication.

Major concerns

1. The HCA dataset lacks ancestry information for ~70% of samples, which fundamentally compromises any statistical claim about over- or under-representation. Tissue-level ancestry estimates derived from such incomplete data are inherently unreliable, yet the authors interpret them as robust signals. Although the manuscript acknowledges this limitation, it does not evaluate whether the conclusions remain stable under such extreme missingness.

2. Single-cell datasets are often generated for specific biological questions, not for population representativeness. Yet the manuscript assumes that all atlases ought to reflect global/disease demographics, which is an oversimplification. Many HCA datasets derive from specific tissue-banking programs with non-random recruitment (organ donors, surgical excess tissue, pediatric samples). HTAN samples reflect the demographics of contributing institutions, which may not mirror SEER (Surveillance, Epidemiology, and End Results) incidence. The manuscript frames these as "biases" but does not distinguish between (a) systematic exclusion and (b) study-purpose-driven sampling.

3. Beyond the major consortia, a vast amount of single-cell data has been generated by independent studies. To the best of current knowledge, the cumulative sample size of these non-consortium datasets far exceeds the combined total of HCA, HTAN, and PsychAD. However, the manuscript does not account for, analyze, or even acknowledge this much larger body of datasets. As

a result, the authors' conclusions -- particularly those regarding global representativeness, ancestry balance, and field-wide sampling practices -- are drawn from a small and non-representative subset of available single-cell resources. Without incorporating or at least contextualizing these non-consortium datasets, the manuscript risks overstating the generality of its claims.

4. The manuscript repeatedly asserts that demographic imbalance in training data "risks embedding inequities into AI foundation models." While conceptually plausible, no empirical evidence is provided. Specifically: (a) there is no audit of existing foundation models (e.g., scGPT or other atlas-scale models); (b) no analysis demonstrating performance differences across ancestry or sex; (c) no test of whether demographic skew induces biased embeddings or misclassification. These claims extend beyond what the presented data can support and may mislead readers.

5. The analysis collapses all Asian populations into a single category and uses UN continental population statistics as proxies for genetic ancestry -- an approach that oversimplifies global population structure to the point of being misleading. Despite acknowledging this limitation, the manuscript continues to draw strong race-comparative conclusions from these coarse classifications.

6. The "Checklist" in Table 2 reads as a set of general recommendations rather than actionable guidance tailored to single-cell technologies. It omits key domain-specific considerations such as tissue dissociation and procurement constraints, donor availability, equitable barcoding strategies, model-training considerations, and metadata ontology standards. The checklist would benefit from being reframed with concrete, field-specific recommendations.

Reviewer 2:

This commentary discusses an important and timely problem: the need to improve equity in genomics data generation, especially in the field of single cell biology. Creating more diverse datasets is essential for building resources that are scientifically strong, clinically meaningful, and representative of global populations. I agree with the authors that improving equity will directly strengthen clinical translation, including biomarker discovery, disease

stratification, and the development of more accurate models for prediction and diagnosis.

Although the authors highlight the value of equitable data, the commentary does not fully consider several important structural, political and economic challenges that make global equity difficult to achieve. These missing perspectives weaken the overall impact of the argument.

1. National Policies and Regulatory Barriers Differ Greatly Across Regions

Each region has its own data governance policies, which makes coordination very challenging. For example, China has generated a very large amount of genomic data but national regulations prevent the sharing of this information outside its borders. This creates an unavoidable imbalance that affects global datasets. Many other countries in Asia, Africa and Latin America face similar policy constraints. It would strengthen the commentary to acknowledge these realities.

2. Existing Global Consortia Remain Centered in Western Countries

Large research efforts such as ENCODE, psychENCODE and even the Human Cell Atlas are primarily funded and directed by institutions in the US. As a result, the majority of samples originate from Western populations. Discussing how US/European policy makers, funding agencies, and philanthropic foundations might support research efforts in other parts of the world could offer meaningful ways to address this imbalance.

3. High Economic Barriers and High Cost of Technology

Relatedly, achieving equity requires more than good intentions. It also depends on financial access to advanced genomics technologies. Single cell sequencing, library preparation kits from commercial providers remain expensive for many laboratories. Without reducing cost or providing financial support, calls for global equity may unintentionally widen existing gaps. This is a conversation that deserves attention in the commentary.

4. Cultural and Community Engagement Is Crucial

True equity requires trust. It depends on respectful engagement with local

communities, clear communication, and cultural understanding. Many populations have historical and religious reasons to be cautious about participation in genomics research. The commentary would benefit from acknowledging the need for community participation and respect for cultural contexts.

5. In the Era of AI, What Does Equity Really Mean?

As AI becomes central to data analysis and interpretation, equity must extend beyond data generation. The majority of AI models and tools are created in Western institutions. This raises concerns about generalizability, bias and the risk of reinforcing existing inequalities. The commentary could explore how equity should be defined when the people who create AI systems do not represent the populations on which these systems will be applied. In other words, un-equitable access to GPUs does not solve the equity issue even if the genomic data is equitable. As an AI group writing this commentary, it will be invaluable to know more about current and future perspectives on this topic.

The commentary in Nature Communications (https://www.nature.com/articles/s41467-024-54306-x) offers a clear and realistic framework for how international consortia can work toward equity. It emphasises governance structures, inclusive funding strategies, data policies that reflect local needs, and long term partnerships across regions. Including insights from such work would greatly enrich the present commentary.

While the authors correctly identify the importance of equity in single cell genomics, the current commentary simplifies many complex issues. It does not fully consider the challenges associated with sample collection, patient recruitment, financial cost, data storage and computational requirements, national data sharing restrictions, community engagement and the broader role of AI in shaping analysis. Therefore, a deeper engagement with the real world limitations would create a more balanced argument for readers of Cell Genomics. Addressing these foundational challenges will help attract broader support globally and provide a clearer roadmap for achieving data and "knowledge" equity.

Reviewer 3:

The manuscript tackles a pressing question: whether global populations are fairly represented in existing single-cell omic atlases. The article draws parallels to similar issues in genome-wide association studies (where Eurocentric data biases have limited broad applicability). However, to maximise impact, the perspective needs to delve deeper into the biological rationale and single-cell-specific consequences of these representation gaps, rather than relying mainly on the GWAS analogy. Currently, the argument appears to rest on an analogy that treats underrepresentation primarily as a fairness or generalizability issue; it would be much stronger if you detail how and why ancestry influences cell biology itself (gene expression, cell states, etc.) and thus why diverse single-cell maps are scientifically critical in their own right.

The writing could also better integrate the current efforts and literature in this area. There are already nascent initiatives and published discussions on diversity in single-cell research (including some the authors cite and others that should be cited; see detailed comments). A reader should come away understanding not just that "current atlases have biases," but that the field acknowledges this and is actively working to fix it - and where your perspective fits into that ongoing movement. Currently, the manuscript only briefly alludes to a few examples; I suggest expanding on them to demonstrate a broader awareness of the landscape. Incorporating these points will not only enrich the perspective but also reassure readers that your calls to action are grounded in (and informed by) what the community is already doing or striving for.

Below, I provide specific suggestions and areas for revision.

Detailed Comments and Suggestions

1. Expand the biological rationale beyond GWAS analogies: The manuscript currently leans heavily on analogies to GWAS diversity issues as justification for why representation matters. While this comparison is a helpful starting point (and indeed the disparities in genomic studies are well-known), it is crucial that you articulate the direct biological reasons why ancestry representation is essential for single-cell atlases. In other words, what are the cellular and molecular consequences of focusing atlas efforts on only a subset of populations? Please consider expanding the discussion along these lines:

a. Ancestry influences on gene expression and cell states: Highlight that genetic ancestry (often correlated with geographic origin) can lead to differences in gene regulatory architecture and expression levels in various cell types. Emerging single-cell studies are beginning to show that many genes have differential expression across ancestries in specific cell types (even among healthy

individuals), often due to a combination of genetic variants and gene-by-environment interactions.

b. Impacts on cell type identification and annotation: A lack of diversity might leave certain cell subtypes undiscovered. Your current text hints at this (by analogy, extrapolating from one population to another is unsound); make it more explicit. This is analogous to how a reference genome that came from a single population missed many genetic variants present elsewhere - except here we risk missing entire cellular phenotypes or wrongly assuming that markers identified in one group are universal.

c. Relevance to disease and therapeutics: You touch on generalizability to disease research; consider giving a concrete example or two (without straying too far into GWAS). For instance, differences in immune cell gene expression across ancestries have been linked to varying susceptibility to autoimmune diseases and to responses to infection. Moreover, drug response can vary with ancestry (one classic example: certain asthma medications like albuterol show reduced efficacy in some African-ancestry populations due to genetic variation affecting receptors).

2. The article repeatedly notes that this is a summary based on self-reported metadata. However, major initiatives are underway that directly estimate genetic ancestral diversity from scRNA-seq data across all samples in major cell atlases. Akin to the assessment of population structure, admixture, and genetic ancestral diversity, this data will provide a substantially advanced version of diversity estimation compared to self-reported labels, as well as being available on ~100% of samples. Releases of this resource are due in Q1 2026, so depending on timing, this review should consider incorporating them. This would help keep the perspective article relevant and avoid it being 'outdated' quickly.

3. Incorporate major diversity-focused initiatives in single-cell research: The perspective would be strengthened by acknowledging and discussing the significant efforts already underway to improve ancestral diversity in single-cell omics. Currently, the manuscript only briefly cites the Human Cell Atlas and mentions diversity, but it doesn't convey the full scope of the community's work. Some initiatives to consider:

a. Human Cell Atlas (HCA) - global diversity efforts: The HCA consortium has made inclusion a priority. You should mention that HCA has an Equity Working Group and several regional networks (Latin America, Africa, Asia, etc.) devoted to ensuring diverse participation and sampling. In 2021, a partnership with the

Chan Zuckerberg Initiative (CZI) launched the Ancestry Networks for the HCA, infusing funding (~$28M) into projects that specifically collect single-cell data from underrepresented populations worldwide. There are now multiple teams globally mapping cells from diverse donors - for example, projects building an African ancestry immune cell atlas, a Latin American cell diversity atlas, an Asian Immune Diversity Atlas, and ancestrally inclusive atlases of specific organs (heart, skin, prostate, etc.).

b. Other consortia and programs: Beyond HCA, mention initiatives like the Human Tumor Atlas Network (HTAN) or disease-focused single-cell consortia that are trying to include diverse cohorts. The Chan Zuckerberg Initiative, in addition to the Ancestry Networks, has funded many single-cell projects in regions outside of North America/Europe and has emphasized diversity in its grant calls.

4. Discuss metadata completeness and ancestry annotation challenges in single-cell data: One practical issue in assessing and improving representation that merits more discussion is the state of metadata in public single-cell resources. As you have likely experienced while compiling data for this study, determining the ancestry or ethnicity of donors in single-cell datasets is not always straightforward; such information is often missing or inconsistently reported. I encourage you to add a section or paragraph acknowledging this problem and describing ongoing efforts to address it:

a. Current metadata gaps: Note that many single-cell studies, especially older or smaller-scale ones, did not include detailed demographic metadata (perhaps assuming it wasn't relevant for a basic cell atlas). Even in large consortium datasets, fields like "donor ancestry" or "ethnic group" are sometimes absent or use non-standardised terms. This lack of metadata makes it difficult to quantify representation or to re-analyze data by population. Without standardised reporting, we may underestimate the diversity that exists in datasets, or, conversely, we may be unable to identify the gaps.

b. Efforts to improve metadata standardization: The community is recognizing this issue. The Human Cell Atlas has a Diversity Task Force (within the Equity Working Group) that examines how to track and encourage reporting of donor diversity in submissions. Data portals and platforms are also working on this - for instance, the CZI's cellxgene platform and the EBI's Single Cell Expression Atlas have begun to incorporate fields for donor ancestry/ethnicity in their metadata schemas. You should discuss how requiring or encouraging authors to provide this information is an important step. It is worth noting that even determining

"ancestry" can be complex, whether through self-identified race/ethnicity or genomic ancestry inferred from genotype data. Both approaches have challenges (self-report can be coarse or socio-politically defined; inference requires genetic data that not all scRNA-seq experiments provide).

5. Examine how study design, funding, and ethics frameworks influence representation: In addition to what the problem is, the perspective should address why we ended up here - and how we can prevent similar biases moving forward. A more reflexive discussion about the systemic factors driving the current lack of diversity would be valuable. Some points to consider including or expanding:

a. Study design and sampling bias: Most single-cell atlas projects to date have drawn samples from readily available sources - often patients or donors local to major research institutions in North America, Europe, and East Asia. Unless a study is intentionally designed to capture diversity, the default is typically whatever is most convenient or prevalent in the biobank. Discuss how choices made in study planning (e.g., selecting cohorts, choosing which tissue samples to prioritise) can unintentionally exclude specific populations. For example, a lab in the US might, without explicit intent, recruit mostly Americans of European descent for a "reference" cell atlas simply because of the hospital's demographics or existing tissue resources.

b. Funding structures and incentives: I feel that it is important to acknowledge the role of funding in shaping representation. Large-scale single-cell projects require considerable resources, and historically, these have been provided by entities in wealthier countries. Until recently, there was little overt requirement from funders that diversity be built into project aims.

c. Ethical and social frameworks: inclusion is not merely a technical issue but an ethical imperative recognised in frameworks such as the Belmont Report principles and recent calls for equity in research. The Human Cell Atlas, for example, held equity roadshows and meetings in regions like Africa and Latin America to engage local scientists and stakeholders. Also, mention any known issues, such as Indigenous data sovereignty or requirements, that might affect how data can be shared internationally. These ethical considerations sometimes explain why certain groups are absent (e.g., due to a lack of appropriate consent to use samples for open-access global atlases).

6. Frame the manuscript's contributions within the broader movement for equitable reference resources: In the conclusion or throughout the discussion, make sure to position your work in the context of the broader push for equity in

biological data resources. Currently, the manuscript presents findings and recommendations, but it would benefit from explicitly linking its advocacy to a broader scientific trend: ensuring that advances benefit all populations, not just a few. A few angles to include:

a. Analogy to the human reference genome efforts: Just as the human genome reference is now being revisited with projects like the Human Pangenome Project to include diverse genomes (because the original reference missed many population-specific variants), the human cell atlas efforts must also build a "pancellular" reference that represents humanity's diversity.

b. Citing the movement in single-cell community: Acknowledge that your article is one of several voices in the community highlighting this issue. For instance, you might note that in addition to new data initiatives, there have been commentaries by others (HCA Equity Working Group's commentary in Nature Medicine 2020 by Majumder et al.)

7. Can you include some references or examples for the statement 'ancestry-associated biology can alter therapeutic options and responses.

8. Can you also provide some evidence or a reference to support that statement 'what if some of the rare and pathogenic cell types are also population specific?'. I am not aware of any work showing cell types being population specific.

Authors' response to the first round of review

Authors (all responses in black): Thank you for the thoughtful evaluation of our manuscript, "Are Different Populations Fairly Represented in Single-Cell Omic Atlases?" We have carefully addressed all reviewer concerns through a substantial revision. Below we provide a detailed point-by-point response, with direct quotations from the revised manuscript where applicable.

We also note that the revised Table 2 addressed many of the reviewers' shared concerns:

Table 2. Practical Checklist for Equitable Single-Cell Omic Study Design

#	KEY QUESTION FOR RESEARCH TEAMS	POSSIBLE SOLUTIONS
**Recruitment & Study Design**		
**1**	Does the planned cohort reflect population incidence/prevalence for the target tissue/disease?	• Set explicit sampling quotas by ancestry<br>• Oversample underrepresented groups
**2**	Is sex balance planned for non–sex-specific tissues/diseases?	• Set sex-stratified quotas
**3**	Have we defined minimum per-group sample sizes to enable stratified analyses by ancestry and/or sex?	• Power analyses by subgroup; enforce thresholds<br>• Phase enrollment until quotas are met
**4**	Are community sites serving underrepresented populations included as recruitment hubs?	• Add community clinics & advisory boards<br>• Culturally tailored outreach & consent
**5**	Are eligibility criteria free of inadvertent exclusions (language, insurance, visit schedule)?	• Translated materials & flexible visit windows<br>• Reimbursements/transport, childcare support
**Pre-Analytical & Single-Cell-Specific**		
**6**	Do tissue procurement and dissociation protocols account for donor availability constraints across sites (e.g., organ-donor programs, surgical schedules)?	• Pre-qualify multiple procurement pipelines per site<br>• Standardize ischemia time, dissociation enzyme/duration, and viability thresholds
**7**	Are biobanking SOPs harmonized across sites (time-to-freeze, buffers, RNase control)?	• Unified SOPs with site audits and QC dashboards<br>• Reject out-of-spec samples uniformly
**8**	Do multiplexing and batch-assignment strategies avoid confounding ancestry/sex with technical batch?	• Randomize or block donors across pools/batches by ancestry and sex<br>• Include cross-site reference samples in every run<br>• Use equitable barcoding allocation (balance hashtag/lipid oligos across groups)
**Metadata & Monitoring**		
**9**	Will ancestry, sex, age, and disease-specific clinical variables be mandatory fields using controlled vocabularies?	• Require fields at upload using ontology standards (e.g., cellxgene schema, EBI SCEA metadata)<br>• Validate against HAncestro for ancestry
**10**	Are missingness dashboards (ancestry × sex × site) reviewed regularly?	• Publish dashboards; open action items to sites when gaps emerge
**11**	Is there a plan for *post-hoc* enrichment if monitoring shows imbalance?	• Pre-specify trigger rules (e.g., <20% of target quota)<br>• Add contingency recruitment sites; targeted outreach
**12**	Will raw + harmonized metadata be versioned and publicly released?	• Robust versioning & changelog on every data release
**Community Engagement & Ethics**		
**13**	Have we planned return-of-value to sustain participation from diverse communities?	• Share lay summaries and participant newsletters<br>• Host community feedback sessions
**14**	Does consent language address data sovereignty, international sharing, and re-use in AI model training?	• Consult local governance frameworks (e.g., CARE Principles for Indigenous data)<br>• Include explicit clauses on computational re-use and AI model training
**Downstream AI/ML Model Development**		
**15**	Will training-data demographic composition be disclosed in model documentation?	• Report ancestry/sex breakdown of all training and test splits<br>• Flag known gaps relative to target application population
**16**	Will model performance be evaluated and balanced when stratified by ancestry and sex?	• Report cell-type annotation accuracy, embedding quality, and cell marker predictions per demographic group<br>• Include robustness tests under simulated demographic shift

Reviewers' Comments (all comments in blue):

Reviewer #1: In this Perspective paper, the authors conduct a secondary analysis of publicly available metadata of large single-cell consortia, including HCA, HTAN, and PsychAD and argue that existing single-cell resources exhibit substantial ancestry and sex biases. The topic may be of broad interest, but in its current form, the manuscript suffers from conceptual oversimplification, methodological opacity, and overstatement of conclusions relative to the evidence provided. Substantial revision and strengthening are required before this work could be suitable for publication.

Authors: We thank the reviewer for this constructive assessment. We have substantially revised the manuscript to deepen the biological rationale, add methodological rigor through sensitivity analyses, temper claims about AI models, and provide field-specific actionable guidance. Each specific concern is addressed below.

Major concerns

1. The HCA dataset lacks ancestry information for ~70% of samples, which fundamentally compromises any statistical claim about over- or under-representation. Tissue-level ancestry estimates derived from such incomplete data are inherently unreliable, yet the authors interpret them as robust signals. Although the manuscript acknowledges this limitation, it does not evaluate whether the conclusions remain stable under such extreme missingness.

Authors: We agree that this level of missingness demands careful treatment before a conclusion can be drawn. In addition to acknowledging this metadata missingness in the discussion, we have added sensitivity analyses for HCA. In the revised manuscript:

> *"Because conclusions drawn from complete-case analysis alone could be severely biased if missingness is non-random, we evaluated the robustness of representation findings. Applying partial-identification bounds (lower bound assuming no missing donors belong to the group; upper bound assuming all missing donors belong to the group)(**Supplementary Table 2**). European over-representation is the only conclusion that is robust to all plausible missing-data mechanisms: even under the most conservative assumption (all 7,082 missing donors are non-European), the European lower bound (17.0%) still exceeds the global reference (9.4%)."*

2. Single-cell datasets are often generated for specific biological questions, not for population representativeness. Yet the manuscript assumes that all atlases ought to reflect global/disease demographics, which is an oversimplification. Many HCA datasets derive from specific tissue-banking programs with non-random recruitment (organ donors, surgical excess tissue, pediatric samples). HTAN samples reflect the demographics of contributing institutions, which may not mirror SEER (Surveillance, Epidemiology, and End Results) incidence. The manuscript frames these as "biases" but does not distinguish between (a) systematic exclusion and (b) study-purpose-driven sampling.

Authors: We now explicitly acknowledge this distinction. In the HCA results section:

> *" These skews in ancestral proportions likely reflect study-purpose-driven sampling rather than intentional exclusion: many HCA datasets derive from tissue-banking programs with non-random recruitment (organ donors, surgical excess, pediatric samples) whose demographics reflect the population served by contributing institutions."*

For HTAN specifically, we also added to the sex analysis:

> *"These sex patterns are sometimes directionally consistent with physiological expectations in sex-specific disease tissues, but also suggest potential ascertainment or study-design bias elsewhere."*

Finally we added to the Discussion,

> *"Third, our analyses of ancestral/sex proportions do not resolve whether the imbalances are a result of study-purpose-driven sampling or intentional/unintentional exclusion."*

3. Beyond the major consortia, a vast amount of single-cell data has been generated by independent studies. To the best of current knowledge, the cumulative sample size of these non-consortium datasets far exceeds the combined total of HCA, HTAN, and PsychAD. However, the manuscript does not account for, analyze, or even acknowledge this much larger body of datasets. As a result, the authors' conclusions -- particularly those regarding global representativeness, ancestry balance, and field-wide sampling practices -- are drawn from a small and non-representative subset of available single-cell resources. Without incorporating or at least contextualizing these non-consortium datasets, the manuscript risks overstating the generality of its claims.

Authors: We now address this prominently in the Limitations section:

> "*Finally, our analyses focused on 3 publicly available single-cell consortium datasets, which may not reflect the demographic composition of all other single-cell omic resources. Non-consortium datasets collectively have exceeded these consortia in sample size and may differ in composition, but often offer even less standardized metadata for analyses. Conclusions herein are based on data from these specific consortia, not the field as a whole.*"

4. The manuscript repeatedly asserts that demographic imbalance in training data "risks embedding inequities into AI foundation models." While conceptually plausible, no empirical evidence is provided. Specifically: (a) there is no audit of existing foundation models (e.g., scGPT or other atlas-scale models); (b) no analysis demonstrating performance differences across ancestry or sex; (c) no test of whether demographic skew induces biased embeddings or misclassification. These claims extend beyond what the presented data can support and may mislead readers.

Authors: We have substantially toned down this claim and reframed it as a structural risk rather than a demonstrated harm. We explicitly acknowledge the absence of empirical benchmarks:

> "*This can lead to challenges when a significant portion of future advances may be driven by AI foundation models built on these skewed datasets. For example, single-cell AI foundation models that are already widely used, such as scGPT[12], Geneformer, and scFoundation maximized the size of their training datasets by combining existing public datasets. Consequently, most foundation models include HCA as one of the major sources and leverage HTAN data if they separately trained a cancer-focused model without, to our knowledge, supplementation of data from underrepresented populations[12-14], thus possibly inheriting the bias from these datasets without most users' awareness. A controlled benchmark of AI model performance stratified by donor ancestry remains needed.*"

5. The analysis collapses all Asian populations into a single category and uses UN continental population statistics as proxies for genetic ancestry -- an approach that oversimplifies global population structure to the point of being misleading. Despite acknowledging this limitation, the manuscript continues to draw strong race-comparative conclusions from these coarse classifications.

Authors: This was addressed with expanded texts in the Limitations:

> "*First, ancestry was analyzed as reported metadata rather than genetically inferred, which may introduce misclassification. Classifying ancestry groups into broad categories like "European" or "Asian" is challenging due to the complex and personal nature of ethnic identity. These broad categories collapse substantial within-group heterogeneity*

and conflate self-identified race/ethnicity with genetic ancestry. Where metadata permitted, we sought finer granularity (see Methods: Curation of Demographic Data in Single-Cell Consortia Studies), but most datasets did not support it. Some groups, such as Ashkenazi Jews, classified as "European," have distinct cultural and genetic backgrounds that may not fit neatly into this category. Similarly, "Hispanic/Latino" encompasses individuals with mixed heritage. "

Now, we also added this prominently in Results,

"Compared to global expectations (Europe 9.4%, Asia 59.4%, Africa 17.6%, Latino 13.6%), Europeans were overrepresented sixfold, while Asians, Africans, and Latinos were consistently underrepresented (0.36×, 0.59×, and 0.45×, respectively; $p < 2e-16$). We acknowledge that the use of the global reference is an oversimplification that provides only coarse categories during the curation process of comparable ancestry metadata (Methods)."

6. The "Checklist" in Table 2 reads as a set of general recommendations rather than actionable guidance tailored to single-cell technologies. It omits key domain-specific considerations such as tissue dissociation and procurement constraints, donor availability, equitable barcoding strategies, model-training considerations, and metadata ontology standards. The checklist would benefit from being reframed with concrete, field-specific recommendations.

Authors: We have completely rewritten the checklist to be field-specific and actionable:

*"To translate this into actionable steps, we developed a field-specific checklist (**Table 2**) covering 16 specific questions and 5 domains across the full study lifecycle: procurement and recruitment planning aligned to sampling frame; pre-analytical considerations including tissue dissociation and donor availability; equitable multiplexing and batch-assignment strategies to avoid confounding demographics with batch effects; minimum required demographic metadata fields and controlled vocabularies (aligned with cellxgene and EBI standards); standardized biobanking protocols; consent language and data-sovereignty considerations for international collaborations; and AI model-development reporting standards including training-data composition disclosure, stratified performance evaluation across ancestry and sex, and robustness testing. Each domain pairs a critical question with concrete solutions—for example, stratified recruitment quotas, community clinic partnerships, and pre-specified minimum sample sizes per ancestry group. This approach aims to address bias proactively, at the point of study design, where correction is most effective."*

Reviewer #2: This commentary discusses an important and timely problem: the need to improve equity in genomics data generation, especially in the field of single cell biology. Creating more diverse datasets is essential for building resources that are scientifically strong, clinically meaningful, and representative of global populations. I agree with the authors that improving equity will directly strengthen clinical translation, including biomarker discovery, disease stratification, and the development of more accurate models for prediction and diagnosis.

Although the authors highlight the value of equitable data, the commentary does not fully consider several important structural, political and economic challenges that make global equity difficult to achieve. These missing perspectives weaken the overall impact of the argument.

Authors: We thank the reviewer for highlighting these critical real-world dimensions and considerations. We have added substantial texts that address each point raised, including the fully revised **Table 2** above.

1. National Policies and Regulatory Barriers Differ Greatly Across Regions

Each region has its own data governance policies, which makes coordination very challenging. For example, China has generated a very large amount of genomic data but national regulations prevent the sharing of this information outside its borders. This creates an unavoidable imbalance that affects global datasets. Many other countries in Asia, Africa and Latin America face similar policy constraints. It would strengthen the commentary to acknowledge these realities.

Authors: We now discuss this explicitly:

> *"The demographic skews emerge from intersecting structural forces. Most atlas projects draw samples from readily available sources at major institutions, often defaulting to local demographics. Funding for large-scale projects has historically come from wealthier countries without explicit diversity requirements. Single-cell technology remains expensive, limiting participation from low-resource settings. National data-governance policies create additional constraints: many countries restrict sharing individual-level genomic data outside their borders. Finally, achieving equity requires respectful community engagement—many populations have historical or cultural reasons for caution, and frameworks such as Indigenous data sovereignty principles appropriately constrain how data can be shared globally."*

2. Existing Global Consortia Remain Centered in Western Countries

Large research efforts such as ENCODE, psychENCODE and even the Human Cell Atlas are primarily funded and directed by institutions in the US. As a result, the majority of samples originate from Western populations. Discussing how US/European policy makers, funding agencies, and philanthropic foundations might support research efforts in other parts of the world could offer meaningful ways to address this imbalance.

Authors: As in the paragraph above we have added:

> *" Funding for large-scale projects has historically come from wealthier countries without explicit diversity requirements. Single-cell technology remains expensive, limiting participation from low-resource settings."*

We also highlight positive developments:

> *"In 2021, CZI launched Ancestry Networks for the HCA funding single-cell data collection from underrepresented populations worldwide, supporting projects including African, Latin American, and Asian immune diversity atlases and ancestrally inclusive organ atlases."*

3. High Economic Barriers and High Cost of Technology

Relatedly, achieving equity requires more than good intentions. It also depends on financial access to advanced genomics technologies. Single cell sequencing, library preparation kits from commercial providers remain expensive for many laboratories. Without reducing cost or providing financial support, calls for global equity may unintentionally widen existing gaps. This is a conversation that deserves attention in the commentary.

Authors: The economic barriers were added to discussion as in response to the point above.

We also added this as one recommendation:

"*Providing technology-access programs in both single-cell and AI technologies is needed to help address such disparity.*"

4. Cultural and Community Engagement Is Crucial

True equity requires trust. It depends on respectful engagement with local communities, clear communication, and cultural understanding. Many populations have historical and religious

reasons to be cautious about participation in genomics research. The commentary would benefit from acknowledging the need for community participation and respect for cultural contexts.
Authors: We acknowledge this important point. In addition to the addition of the "Community Engagement" section in **Table 2** (see above), we have also now added in text:

> *"Finally, achieving equity requires respectful community engagement—many populations have historical or cultural reasons for caution, and frameworks such as Indigenous data sovereignty principles appropriately constrain how data can be shared."*

5. In the Era of AI, What Does Equity Really Mean?
As AI becomes central to data analysis and interpretation, equity must extend beyond data generation. The majority of AI models and tools are created in Western institutions. This raises concerns about generalizability, bias and the risk of reinforcing existing inequalities. The commentary could explore how equity should be defined when the people who create AI systems do not represent the populations on which these systems will be applied. In other words, un-equitable access to GPUs does not solve the equity issue even if the genomic data is equitable. As an AI group writing this commentary, it will be invaluable to know more about current and future perspectives on this topic.
Authors: We have added texts that address this point:

> *"A controlled benchmark of AI model performance stratified by donor ancestry remains needed. Moreover, most AI tools are developed in Western institutions with GPU infrastructure; researchers in low- and middle-income countries may lack resources to run these models, creating a secondary inequity where analytical tools—not just reference data—reflect a narrow perspective. Providing technology-access programs in both single-cell and AI technologies is needed to help address such disparity."*

The commentary in Nature Communications (https://www.nature.com/articles/s41467-024-54306-x) offers a clear and realistic framework for how international consortia can work toward equity. It emphasises governance structures, inclusive funding strategies, data policies that reflect local needs, and long term partnerships across regions. Including insights from such work would greatly enrich the present commentary.

While the authors correctly identify the importance of equity in single cell genomics, the current commentary simplifies many complex issues. It does not fully consider the challenges associated with sample collection, patient recruitment, financial cost, data storage and computational requirements, national data sharing restrictions, community engagement and the broader role of AI in shaping analysis. Therefore, a deeper engagement with the real world limitations would create a more balanced argument for readers of Cell Genomics. Addressing these foundational challenges will help attract broader support globally and provide a clearer roadmap for achieving data and "knowledge" equity.
Authors: In addition to using Amit et al. 2024 as the first reference to describe the HCA, we have now cited this useful reference more times, as the framework this commentary provides is extremely relevant and helpful. For example,

> *"A recent framework from the HCA outlines governance, inclusive funding, and cross-regional partnership principles for equitable consortia[1]."*

We thank the reviewer for providing such insightful comments. We have also updated **Table 2** significantly (above) to reflect considerations of real-world limitations and hoping that this can help researchers design better scOmics studies even under such constraints.

Reviewer #3: The manuscript tackles a pressing question: whether global populations are fairly represented in existing single-cell omic atlases. The article draws parallels to similar issues in genome-wide association studies (where Eurocentric data biases have limited broad applicability). However, to maximise impact, the perspective needs to delve deeper into the biological rationale and single-cell-specific consequences of these representation gaps, rather than relying mainly on the GWAS analogy. Currently, the argument appears to rest on an analogy that treats underrepresentation primarily as a fairness or generalizability issue; it would be much stronger if you detail how and why ancestry influences cell biology itself (gene expression, cell states, etc.) and thus why diverse single-cell maps are scientifically critical in their own right.
The writing could also better integrate the current efforts and literature in this area. There are already nascent initiatives and published discussions on diversity in single-cell research (including some the authors cite and others that should be cited; see detailed comments). A reader should come away understanding not just that "current atlases have biases," but that the field acknowledges this and is actively working to fix it - and where your perspective fits into that ongoing movement. Currently, the manuscript only briefly alludes to a few examples; I suggest expanding on them to demonstrate a broader awareness of the landscape. Incorporating these points will not only enrich the perspective but also reassure readers that your calls to action are grounded in (and informed by) what the community is already doing or striving for.
Authors: We thank the reviewer for this guidance. We have added several new paragraphs that provide the biological depth requested. Each detailed comment is addressed below.

Below, I provide specific suggestions and areas for revision.
Detailed Comments and Suggestions
1. Expand the biological rationale beyond GWAS analogies: The manuscript currently leans heavily on analogies to GWAS diversity issues as justification for why representation matters. While this comparison is a helpful starting point (and indeed the disparities in genomic studies are well-known), it is crucial that you articulate the direct biological reasons why ancestry representation is essential for single-cell atlases. In other words, what are the cellular and molecular consequences of focusing atlas efforts on only a subset of populations? Please consider expanding the discussion along these lines:
a. Ancestry influences on gene expression and cell states: Highlight that genetic ancestry (often correlated with geographic origin) can lead to differences in gene regulatory architecture and expression levels in various cell types. Emerging single-cell studies are beginning to show that many genes have differential expression across ancestries in specific cell types (even among healthy individuals), often due to a combination of genetic variants and gene-by-environment interactions.
b. Impacts on cell type identification and annotation: A lack of diversity might leave certain cell subtypes undiscovered. Your current text hints at this (by analogy, extrapolating from one population to another is unsound); make it more explicit. This is analogous to how a reference genome that came from a single population missed many genetic variants present elsewhere - except here we risk missing entire cellular phenotypes or wrongly assuming that markers identified in one group are universal.
c. Relevance to disease and therapeutics: You touch on generalizability to disease research; consider giving a concrete example or two (without straying too far into GWAS). For instance, differences in immune cell gene expression across ancestries have been linked to varying susceptibility to autoimmune diseases and to responses to infection. Moreover, drug response can vary with ancestry (one classic example: certain asthma medications like albuterol show reduced efficacy in some African-ancestry populations due to genetic variation affecting receptors).

Authors: We thank the reviewer for this careful comment, we have now added multiple sections to address this, particularly:
1a. Ancestry influences on gene expression and cell states & 1b. Impacts on cell type identification and annotation.
We now provide specific single-cell evidence where ancestry influence single-cell gene expression and cell states. We also use the pancellular reference and point to the challenge of missing cell subtypes or finding the wrong cell marker. This is now added to the Introduction:

> *"Genetic ancestry shapes cell-type-specific gene expression and regulatory architecture. Single-cell studies of viral immune responses have identified hundreds of genes with ancestry-differential expression in specific immune cell subsets[12,13], and patient ancestry modulates innate immune activation at the level of individual cell states in SARS-CoV-2 responses[14]. An atlas from one ancestral background therefore risks missing cell subtypes or wrongly assuming universal cell markers—paralleling how the original reference genome missed variants or genome segments now recovered by the Human Pangenome Project[15]. While definitive population-specific cell types or cell states remain to be proven, many pathogenic variants are unique to specific populations[16,17], and ancestry-driven regulatory variation can shift cell-state in disease-relevant cell populations that a homogeneous atlas would fail to catalog. Diverse single-cell maps are therefore a prerequisite for biological completeness—a pancellular reference that, like the pangenome, must represent humanity's full diversity to be scientifically universal[18,19]."*

1c. Relevance to disease and therapeutics.
We provide the concrete pharmacogenomic examples requested in Discussion, while acknowledge that single-cell signatures are not yet directly used for most clinical stratification:

> *"This is not a trivial concern: genetic background influences gene expression and regulatory variation[23], sex differences shape immune responses and disease trajectories[24], and ancestry-associated biology can alter therapeutic options and responses, e.g., in genetics, CYP2D6 allele frequencies governing metabolism of ~25% of clinical drugs differ markedly across populations [25,26]. While single-cell signatures have yet been widely used for clinical stratification, over-representation of Europeans in single-cell genomics precludes discovery of biology that exists across the wide spectrum of human diversity and risks building single-cell models that obscure the cellular mechanisms most relevant to underrepresented groups."*

2. The article repeatedly notes that this is a summary based on self-reported metadata. However, major initiatives are underway that directly estimate genetic ancestral diversity from scRNA-seq data across all samples in major cell atlases. Akin to the assessment of population structure, admixture, and genetic ancestral diversity, this data will provide a substantially advanced version of diversity estimation compared to self-reported labels, as well as being available on ~100% of samples. Releases of this resource are due in Q1 2026, so depending on timing, this review should consider incorporating them. This would help keep the perspective article relevant and avoid it being 'outdated' quickly.
Authors: We thank the reviewer for this helpful insight. We have now added this text to Discussion.

> *"Technically, initiatives to estimate genetic ancestry directly from scRNA-seq data via SNP-based approaches[32] may provide ancestry estimates for nearly all atlas samples, superseding reliance on self-reported labels. Data portals, including cellxgene and EBI's Single Cell Expression Atlas are incorporating structured ancestry fields in metadata schemas."*

However, we assessed the cellxgene API and did not find updated ancestry inference label (as of March 4th 2026). Thus, we did not provide an updated analyses based on that data. We also did add a sensitivity analysis for HCA in the results section

> *"Because conclusions drawn from complete-case analysis alone could be severely biased if missingness is non-random, we evaluated the robustness of representation findings. Applying partial-identification bounds (lower bound assuming no missing donors belong to the group; upper bound assuming all missing donors belong to the group)(**Supplementary Table 2**). European over-representation is the only conclusion that is robust to all plausible missing-data mechanisms: even under the most conservative assumption (all 7,082 missing donors are non-European), the European lower bound (17.0%) still exceeds the global reference (9.4%)."*

Thus, the conclusion is more robust independent of how the missing data.

3. Incorporate major diversity-focused initiatives in single-cell research: The perspective would be strengthened by acknowledging and discussing the significant efforts already underway to improve ancestral diversity in single-cell omics. Currently, the manuscript only briefly cites the Human Cell Atlas and mentions diversity, but it doesn't convey the full scope of the community's work. Some initiatives to consider:
a. Human Cell Atlas (HCA) - global diversity efforts: The HCA consortium has made inclusion a priority. You should mention that HCA has an Equity Working Group and several regional networks (Latin America, Africa, Asia, etc.) devoted to ensuring diverse participation and sampling. In 2021, a partnership with the Chan Zuckerberg Initiative (CZI) launched the Ancestry Networks for the HCA, infusing funding (~$28M) into projects that specifically collect single-cell data from underrepresented populations worldwide. There are now multiple teams globally mapping cells from diverse donors - for example, projects building an African ancestry immune cell atlas, a Latin American cell diversity atlas, an Asian Immune Diversity Atlas, and ancestrally inclusive atlases of specific organs (heart, skin, prostate, etc.).
b. Other consortia and programs: Beyond HCA, mention initiatives like the Human Tumor Atlas Network (HTAN) or disease-focused single-cell consortia that are trying to include diverse cohorts. The Chan Zuckerberg Initiative, in addition to the Ancestry Networks, has funded many single-cell projects in regions outside of North America/Europe and has emphasized diversity in its grant calls.

Authors: We thank the reviewer for providing these useful insights. Based on this, we have now added this text to the discussion,

> *""The research community is actively addressing these gaps. The HCA Equity Working Group and Diversity Task Force track and encourage donor diversity reporting[30]. In 2021, CZI launched Ancestry Networks for the HCA funding single-cell data collection from underrepresented populations worldwide, supporting projects including African, Latin American, and Asian immune diversity atlases and ancestrally inclusive organ atlases[31]. A recent framework from the HCA outlines governance, inclusive funding, and cross-regional partnership principles for equitable consortia[1]. Technically, initiatives to estimate genetic ancestry directly from scRNA-seq data via SNP-based approaches[32] may provide ancestry estimates for nearly all atlas samples, superseding reliance on self-reported labels. Data portals, including cellxgene and EBI's Single Cell Expression Atlas are incorporating structured ancestry fields in metadata schemas."*

4. Discuss metadata completeness and ancestry annotation challenges in single-cell data: One practical issue in assessing and improving representation that merits more discussion is the state of metadata in public single-cell resources. As you have likely experienced while compiling data for this study, determining the ancestry or ethnicity of donors in single-cell datasets is not always straightforward; such information is often missing or inconsistently reported. I encourage

you to add a section or paragraph acknowledging this problem and describing ongoing efforts to address it:
a. Current metadata gaps: Note that many single-cell studies, especially older or smaller-scale ones, did not include detailed demographic metadata (perhaps assuming it wasn't relevant for a basic cell atlas). Even in large consortium datasets, fields like "donor ancestry" or "ethnic group" are sometimes absent or use non-standardised terms. This lack of metadata makes it difficult to quantify representation or to re-analyze data by population. Without standardised reporting, we may underestimate the diversity that exists in datasets, or, conversely, we may be unable to identify the gaps.
b. Efforts to improve metadata standardization: The community is recognizing this issue. The Human Cell Atlas has a Diversity Task Force (within the Equity Working Group) that examines how to track and encourage reporting of donor diversity in submissions. Data portals and platforms are also working on this - for instance, the CZI's cellxgene platform and the EBI's Single Cell Expression Atlas have begun to incorporate fields for donor ancestry/ethnicity in their metadata schemas. You should discuss how requiring or encouraging authors to provide this information is an important step. It is worth noting that even determining "ancestry" can be complex, whether through self-identified race/ethnicity or genomic ancestry inferred from genotype data. Both approaches have challenges (self-report can be coarse or socio-politically defined; inference requires genetic data that not all scRNA-seq experiments provide).

Authors: We have addressed this important point on missing metadata and metadata curation throughout, most directly we have added this in Discussion:

> *"This study has several limitations. First, ancestry was analyzed as reported metadata rather than genetically inferred, which may introduce misclassification. Classifying ancestry groups into broad categories like "European" or "Asian" is challenging due to the complex and personal nature of ethnic identity. These broad categories collapse substantial within-group heterogeneity and conflate self-identified race/ethnicity with genetic ancestry. Where metadata permitted, we sought finer granularity (see Methods: Curation of Demographic Data in Single-Cell Consortia Studies), but most datasets did not support it. Some groups, such as Ashkenazi Jews, classified as "European," have distinct cultural and genetic backgrounds that may not fit neatly into this category. Similarly, "Hispanic/Latino" encompasses individuals with mixed heritage."*

In Methods, we describe the tedious process of finding (e.g., for HCA studies, first through the portal then through each study's publication Supplementary information/data) and standardizing metadata:

> *"Curation of Demographic Data in Single-Cell Consortia Studies*
> *Human Cell Atlas (HCA)*
>
> ***Data Source***
>
> *We used publicly available datasets from the Human Cell Atlas (HCA) Bionetwork[1-3] to assess ethnic disparities in single-cell genomics research. The analysis covered datasets from 14 HCA networks, including: adipose, breast, development, eye, gut, heart, immune, kidney, liver, musculoskeletal, nervous system, oral and craniofacial, pancreas, reproduction, and skin. Two networks, genetic diversity and organoid, did not have available datasets and were excluded from this analysis.*
>
> ***Dataset Selection and Metadata Extraction***
>
> *For each network, we accessed all available datasets to review the associated metadata files, typically provided in Excel spreadsheet format. These metadata files were examined to identify clinical and demographic information about the donors, specifically focusing on age, gender, ethnicity, and disease status. If demographic metadata was present, we extracted and compiled the relevant information into a single comprehensive spreadsheet for each network.*

In cases where ethnicity data was absent from the metadata, we manually reviewed the publications associated with the dataset. These publications were linked within each dataset's page and often contained supplementary information. Ethnicity data was most frequently found in the Materials and Methods sections or the supplementary materials of these publications. We documented the presence or absence of metadata for each dataset and categorized the ethnicity reporting as follows:
1. Metadata Availability: We recorded whether the study included metadata with clinical and demographic data.
2. Ethnicity Reporting in Metadata: If ethnicity was present in the metadata, we marked it as reported.
3. Ethnicity Reporting in Publications: If no ethnicity data was found in the metadata, we documented whether it was available in the publication.
If ethnicity was provided in the metadata, the column asking about ethnicity data in publications was marked as "N/A." In cases where the ethnicity data in the publication were incomplete or unclear (e.g., presented as proportions or graphs without clear detail), we flagged this limitation with an asterisk.
Standardization of Ancestry and Ethnicity Categories
Given the variability in how ethnicity data were reported across projects (e.g., "Asian," "East Asian," or "Korean"), we standardized the ethnicity categories to allow for consistent comparisons across datasets. The following broad categories were used:

- *European*
- *African*
- *East Asian*
- *South Asian*
- *Asian: For cases where further specification was not provided.*
- *American: Including Hispanic/Latino and Native American individuals.*
- *Other: Encompassing other ethnic groups not covered by the categories above and individuals of mixed ethnic backgrounds.*
- *Unknown*

All Asian categories were eventually merged in analyses due to the constraint from some studies. All more granular data were also preserved in the data available for download in this manuscript.
Donors from other species (e.g., Mus musculus) and cell lines, including human-induced pluripotent stem cells (iPSCs), were excluded from the dataset."

4b. Efforts to improve metadata standardization (cellxgene, EBI, HCA Diversity Task Force).
We thank the reviewer to point out these efforts and have now added this:

> *"Data portals including cellxgene and EBI's Single Cell Expression Atlas are incorporating structured ancestry fields in metadata schemas."*

And in recommendations:

> *"…minimum required demographic metadata fields and controlled vocabularies (aligned with cellxgene and EBI standards)… (**Table 2 above**)"*

We recommend following these standards to address the metadata standardization challenge described by the reviewer.

5. Examine how study design, funding, and ethics frameworks influence representation: In addition to what the problem is, the perspective should address why we ended up here - and

how we can prevent similar biases moving forward. A more reflexive discussion about the systemic factors driving the current lack of diversity would be valuable. Some points to consider including or expanding:

a. Study design and sampling bias: Most single-cell atlas projects to date have drawn samples from readily available sources - often patients or donors local to major research institutions in North America, Europe, and East Asia. Unless a study is intentionally designed to capture diversity, the default is typically whatever is most convenient or prevalent in the biobank. Discuss how choices made in study planning (e.g., selecting cohorts, choosing which tissue samples to prioritise) can unintentionally exclude specific populations. For example, a lab in the US might, without explicit intent, recruit mostly Americans of European descent for a "reference" cell atlas simply because of the hospital's demographics or existing tissue resources.

b. Funding structures and incentives: I feel that it is important to acknowledge the role of funding in shaping representation. Large-scale single-cell projects require considerable resources, and historically, these have been provided by entities in wealthier countries. Until recently, there was little overt requirement from funders that diversity be built into project aims.

c. Ethical and social frameworks: inclusion is not merely a technical issue but an ethical imperative recognised in frameworks such as the Belmont Report principles and recent calls for equity in research. The Human Cell Atlas, for example, held equity roadshows and meetings in regions like Africa and Latin America to engage local scientists and stakeholders. Also, mention any known issues, such as Indigenous data sovereignty or requirements, that might affect how data can be shared internationally. These ethical considerations sometimes explain why certain groups are absent (e.g., due to a lack of appropriate consent to use samples for open-access global atlases).

Authors:

5a. Study design and sampling bias.

We have addressed in the structural barriers section:

> *"Most atlas projects draw samples from readily available sources at major institutions, defaulting to local demographics unless diversity is considered during study design."*

5b. Funding structures and incentives.

We have now added:

> *" Funding for large-scale projects has historically come from wealthier countries without explicit diversity requirements. Large research institutions that conduct such studies may also concentrate on certain geographical regions where recruited samples do not reflect the diversity of national or global populations."*

5c. Ethical and social frameworks; Indigenous data sovereignty.

We have added other ongoing diversity efforts in single cell (in response to R3's comment #3). We have also added these texts to recognize policies and frameworks that may affect how data can be shared:

> *" National data-governance policies create additional constraints: many countries restrict sharing individual-level genomic data outside their borders. Finally, achieving equity requires respectful community engagement—many populations have historical or cultural reasons for caution, and frameworks such as Indigenous data sovereignty principles[29] appropriately constrain how data can be shared."*

6. Frame the manuscript's contributions within the broader movement for equitable reference resources: In the conclusion or throughout the discussion, make sure to position your work in the context of the broader push for equity in biological data resources. Currently, the manuscript presents findings and recommendations, but it would benefit from explicitly linking its advocacy to a broader scientific trend: ensuring that advances benefit all populations, not just a few. A few angles to include:

a. Analogy to the human reference genome efforts: Just as the human genome reference is now being revisited with projects like the Human Pangenome Project to include diverse genomes (because the original reference missed many population-specific variants), the human cell atlas efforts must also build a "pancellular" reference that represents humanity's diversity.
b. Citing the movement in single-cell community: Acknowledge that your article is one of several voices in the community highlighting this issue. For instance, you might note that in addition to new data initiatives, there have been commentaries by others (HCA Equity Working Group's commentary in Nature Medicine 2020 by Majumder et al.)

Authors: We thank the reviewer for this wonderful suggestion, and make pan-cellular a recurring framing throughout the manuscript:

> *"Diverse single-cell maps are therefore a prerequisite for biological completeness—a pancellular reference that, like the pangenome, must represent humanity's full diversity to be scientifically universal[18,19]."*

And in the conclusion:

> *"A pancellular reference representing the full spectrum of human ancestry, sex, and environmental exposure is the only kind of atlas that can claim to be a universal map of human biology."*

6b. We have now cited the Majumder et al. HCA Equity Working Group commentary among many others that are actively addressing this:

> *"The research community is actively addressing these gaps. The HCA Equity Working Group and Diversity Task Force track and encourage donor diversity reporting[30]. In 2021, CZI launched Ancestry Networks for the HCA funding single-cell data collection from underrepresented populations worldwide, supporting projects including African, Latin American, and Asian immune diversity atlases and ancestrally inclusive organ atlases[31]. A recent framework from the HCA outlines governance, inclusive funding, and cross-regional partnership principles for equitable consortia[1]. Technically, initiatives to estimate genetic ancestry directly from scRNA-seq data via SNP-based approaches[32] may provide ancestry estimates for nearly all atlas samples, superseding reliance on self-reported labels. Data portals, including cellxgene and EBI's Single Cell Expression Atlas are incorporating structured ancestry fields in metadata schemas."*

7. Can you include some references or examples for the statement 'ancestry-associated biology can alter therapeutic options and responses.

Authors: We have added this to illustrate "ancestry-associated biology can alter therapeutic options and responses." As there are many cases of this for genetics, we chose one that affects a significant range of drugs/conditions:

> *"This is not a trivial concern: genetic background influences gene expression and regulatory variation[23], sex differences shape immune responses and disease trajectories[24], and ancestry-associated biology can alter therapeutic options and responses, e.g., in genetics, CYP2D6 allele frequencies governing metabolism of ~25% of clinical drugs differ markedly across populations [25,26]."*

8. Can you also provide some evidence or a reference to support that statement 'what if some of the rare and pathogenic cell types are also population specific?'. I am not aware of any work showing cell types being population specific.

Authors: We have deleted the original question. We also reframed this claim to be appropriately cautious in Introduction while providing supporting evidence that address the reviewer's other comments:

> *" Genetic ancestry shapes cell-type-specific gene expression and regulatory architecture. Single-cell studies of viral immune responses have identified hundreds of*

> *genes with ancestry-differential expression in specific immune cell subsets[12,13], and patient ancestry modulates innate immune activation at the level of individual cell states in SARS-CoV-2 responses[14]. An atlas from one ancestral background therefore risks missing cell subtypes or wrongly assuming universal cell markers—paralleling how the original reference genome missed variants or genome segments now recovered by the Human Pangenome Project[15]. While definitive population-specific cell types or cell states remain to be proven, many pathogenic variants are unique to specific populations[16,17], and ancestry-driven regulatory variation can shift cell-state in disease-relevant cell populations that a homogeneous atlas would fail to catalog. Diverse single-cell maps are therefore a prerequisite for biological completeness—a pancellular reference that, like the pangenome, must represent humanity's full diversity to be scientifically universal[18,19]."*

This acknowledges the reviewer's point that strictly population-specific cell types have not yet been demonstrated, while articulating the plausible biological mechanism through which ancestry-driven regulatory variation could produce population-enriched rare cell states.

Referees' report, second round of review

Reviewer 1:

The authors have well answered my concerns. I have no further comments.

Reviewer 3:

Comments enter in Overall, this revision represents a substantive and conscientious response to the concerns raised in Round 1. Key changes related to the 1st round of feedback are an expansion of the biological rationale with single-cell-specific evidence, sensitivity analyses to address the missing-data problem in HCA, a much-improved discussion of structural barriers and community initiatives, and a better framework covering 16 questions across 5 domains.

There are a few small issues that, if addressed, would strengthen the work.

1. Incomplete sentence in the Abstract: "We contextualize these findings within the structural, economic, and regulatory, survey the growing ecosystem…"

2. The sensitivity analysis using partial-identification bounds strengthens the HCA conclusions. However, the concept is introduced with minimal explanation and is likely unfamiliar to many readers. A bridging sentence that explains what the bounds intuitively represent would help.

3. Table 2 is now substantially better than the original checklist. But items 15 and 16 (AI/ML model development reporting) sit somewhat disconnectedly from the

wet-lab and recruitment items that precede them. A brief bridging sentence in the main text explaining why these downstream AI considerations belong within a single-cell study design checklist (rather than in a separate AI development framework) would improve the logical flow

4. Typo: 'HCA Ethnics Working Group'

this field will be shared with the author; your identity will remain anonymous.

Authors’ response to the second round of review

Response to Reviewers

Manuscript: "Are Different Populations Fairly Represented in Single-Cell Omic Atlases?"

We thank the reviewers for their careful and constructive evaluation of our revised manuscript. We address each remaining point below. Reviewer comments are reproduced verbatim in italics; our responses and the corresponding manuscript changes follow.

Reviewer #1

> *"The authors have answered my concerns well. I have no further comments."*

Authors: We thank Reviewer #1 for their positive assessment and constructive feedback in the first round, which substantially improved the manuscript.

Reviewer #3

> *"Overall, this revision represents a substantive and conscientious response to the concerns raised in Round 1. Key changes related to the 1st round of feedback are an expansion of the biological rationale with single-cell-specific evidence, sensitivity analyses to address the missing-data problem in HCA, a much-improved discussion of structural barriers and community initiatives, and a better framework covering 16 questions across 5 domains. There are a few small issues that, if addressed, would strengthen the work."*

Authors: We thank Reviewer #3 for the generous overall assessment and for the four targeted suggestions, each of which we have implemented as described below.

> *"Incomplete sentence in the Abstract: "We contextualize these findings within the structural, economic, and regulatory, survey the growing ecosystem…""*

Authors: We thank the reviewer for catching this. We ensure the full sentence is present in all documents included the tracked/clean word doc and the submission portal and confirm it's within the word limit in Abstract, "We contextualize these findings within the structural, economic, and regulatory, survey the growing ecosystem of diversity-focused initiatives, and provide an actionable, field-specific checklist to help research teams design single-cell studies whose benefits extend equitably across populations."

> *"The sensitivity analysis using partial-identification bounds strengthens the HCA conclusions. However, the concept is introduced with minimal explanation and is likely unfamiliar to many readers. A bridging sentence that explains what the bounds intuitively represent would help."*

Authors: We agree that the concept warrants an intuitive explanation before the technical definition. We have added a bridging sentence that explains the bounds in plain terms:

"Because conclusions drawn from complete-case analysis alone could be severely biased if missingness is non-random, **we considered a simple thought experiment: how would the**

observed European share change at two extremes: if every unlabeled donor turned out to be European (pushing the share to its highest possible value, or ceiling), versus if none of them were European (pushing it to its lowest possible value, or floor)? The true share has to lie somewhere between these two extremes, regardless of why annotations are missing. We refer to this approach as partial-identification bounds, where the upper bound (ceiling) assumes all missing donors belong to the group of interest and the lower bound (floor) assumes none do (Supplementary Table 2)."

> *"Table 2 is now substantially better than the original checklist. But items 15 and 16 (AI/ML model development reporting) sit somewhat disconnectedly from the wet-lab and recruitment items that precede them. A brief bridging sentence in the main text explaining why these downstream AI considerations belong within a single-cell study design checklist (rather than in a separate AI development framework) would improve the logical flow."*

Authors: We appreciate this suggestion and agree that the rationale for embedding AI/ML reporting alongside upstream wet-lab and recruitment items should be made explicit. We have added a bridging sentence in the main text that justifies their inclusion: "…and AI model-development reporting standards including training-data composition disclosure, stratified performance evaluation across ancestry and sex, and robustness testing. **We include the AI/ML model-development items within this single-cell checklist because cellular reference atlases and the AI foundation models trained on them are now produced as a continuous pipeline: the same study-design choices that determine donor diversity at recruitment propagate directly into the training corpora of downstream cell-state classifiers and foundation models. Embedding these considerations within one checklist clarifies accountability.** Each domain pairs a critical question with concrete solutions…"

> *"Typo: 'HCA Ethnics Working Group'"*

Authors: We thank the reviewer for catching this typographical error. "Ethnics" has been corrected to "Ethics."
